# Relationship between Prices and Quality of Essential Medicines from Different Manufacturers Collected in Cameroon, the Democratic Republic of the Congo, and Nigeria

**DOI:** 10.4269/ajtmh.24-0309

**Published:** 2024-10-08

**Authors:** Julia Gabel, Difäm-EPN Minilab Network, Peter Martus, Lutz Heide

**Affiliations:** ^1^ Pharmaceutical Institute, Eberhard Karls University of Tübingen, Tübingen, Germany;; ^2^ Difäm–Ecumenical Pharmaceutical Network Minilab Network, Tübingen, Germany;; ^3^ Institute for Medical Biometrics and Clinical Epidemiology, University Hospital Tübingen, Tübingen, Germany

## Abstract

Achieving universal access to affordable medicines and at the same time ensuring the quality of medicines presents a challenge, especially in low- and middle-income countries. Here, the relationship between medicine prices and medicine quality was investigated in three African countries. From different types of health facilities and medicine vendors, 711 samples of 18 different essential medicines were purchased and analyzed for quality (assay and dissolution) according to the United States Pharmacopeia. Without exception, all originator brand medicines and all SRA generics (generic medicines manufactured in countries with stringent regulatory authorities [SRAs]) complied with pharmacopeial specifications. In contrast, 21.1% of the non-SRA generics (manufactured in countries without SRAs) were substandard. The median prices of originator brands and SRA generics were three times and two times higher than those of non-SRA generics, respectively. Within the non-SRA generics, no positive correlation was observed between medicine quality and medicine price. Medicines manufactured in India, China, or African countries showed similar quality and similar prices. Only a single WHO-prequalified medicine sample was found among the 711 samples. Non-SRA generic medicines produced by manufacturers for which WHO had published Public Inspection Reports showed a significantly lower rate of substandard medicines (7.3%) and, at the same time, significantly lower prices (by 33%) than other non-SRA generics. Falsified medicines (total 2.0%) were found among all categories of medicines and had prices similar to those of non-SRA generics. Our findings indicate that adequate quality assurance does not necessarily imply an increase in medicine prices.

## INTRODUCTION

The WHO has found that on average 10.5% of all medicines in low- and middle-income countries (LMICs) are substandard or falsified (SF),^1^ and two recent systematic reviews have reported even higher percentages.^2^^,^^3^ The WHO estimates that the use of SF medicines amounts to 72,000–169,000 deaths per year from childhood pneumonia globally and to 31,000–116,000 deaths from malaria in sub-Saharan Africa.^1^ Obviously, even much higher numbers are expected if all diseases are considered. Therefore, ensuring the quality of medicines supplied at affordable prices is of prime importance for health care in LMICs.

A considerable body of research has been published on the prices of medicines available from different sources in LMICs.^4^^–^^7^ Furthermore, economic studies have been published on the relationship between price and quality for different goods.^8^^–^^10^ However, despite the public health importance of the subject, there is surprisingly little published evidence on the relationship between medicine prices and medicine quality in LMICs.

Two studies by Bate et al., published in 2011^11^ and 2015,^12^ tried to address this relationship. Both studies were carried out with a similar methodology, collecting medicines from pharmacies in 17 or 18 low-income and middle-income countries, respectively. Using regression analysis and controlling for factors related to medicine type and collection site, the authors estimated that poor-quality samples were on average 13.6–18.7% cheaper than good-quality medicines in their first study and 10% cheaper (95% CI: 7–12%) in their second study. “Falsified” medicines, defined in these studies as medicines not containing the declared active pharmaceutical ingredient (API), were not significantly cheaper than good-quality ones. The value of these studies is limited, however, by the fact that the authors used only simple medicine quality-screening technologies (i.e., thin-layer chromatographic analysis with the Global Pharma Health Fund (GPHF)-Minilab®^13^ and, in their first study, Raman spectroscopy). These methods can reliably detect medicines that do not contain the declared API at all, but they fail to detect a major part of substandard medicines, which contain insufficient amounts of API or show an insufficient dissolution of the API.^1^^,^^14^^–^^16^

Ochekpe et al.^17^ investigated the correlation of price and quality in 71 antimalarial medicine samples collected in Nigeria. An arbitrary method was used to convert the qualitative GPHF-Minilab results into quantitative score values for medicine quality. No significant price difference between high-quality and low-quality medicines was observed.

Also Singal et al.^18^ and Pisani et al.^19^ attempted to evaluate the relationship between medicine prices and medicine quality in India and Indonesia, respectively. Both studies investigated five different medicine types and analyzed assay (amount of the APIs) and dissolution. Pisani et al.^19^ included 204 samples; Singal et al.^18^ did not clearly state the number of samples investigated. However, both studies found that all their investigated samples complied with pharmacopeial specifications. Therefore, although considerable variations in price were observed and evaluated, no variations in quality were found, and neither of the two studies could establish any relationship between price and quality.

Rahman et al.^20^ collected 372 samples of 12 types of medicines in Cambodia; 23.4% of these did not comply with the pharmacopeial specifications for API content, for dissolution, and/or for content uniformity. Noncompliant samples were in some cases cheaper and in other cases more expensive than compliant ones, and no clear picture emerged on the relationship between medicine quality and prices. Likewise, Schiavetti et al.^21^ investigated 239 samples of three types of medicines purchased from private wholesalers in the Democratic Republic of the Congo (DRC); 27.2% of these did not comply with the specifications of the United States Pharmacopeia (USP), mostly owing to an insufficient API content. No correlation was found between quality and price.

In a note added during revision of this manuscript, one reviewer alerted us to a preprint of a further study^22^ not yet listed in PubMed or Web of Science. Also in that study, no relationship between medicine prices and quality was found.

In a small medicine quality study conducted by our own group in Togo (92 samples),^23^ medicines with higher prices showed a lower percentage of samples that were substandard in assay and/or dissolution, but the difference was not statistically significant.

More recently, our group conducted medicine quality studies in Cameroon and the DRC^15^ and in Nigeria.^24^ In collaboration with local faith-based organizations, medicines were purchased from different types of medicine vendors and health facilities and were investigated for assay and dissolution according to the USP. Data on prices and quality were obtained for 711 medicine samples, representing 399 different brands/products and including 14 (2.0%) falsified samples and 132 (18.6%) substandard samples, with different degrees of severity of the observed quality deviations. Detailed results of these medicine quality investigations have been published,^15^^,^^24^^,^^25^ as well as an analysis of the availability and affordability of the medicines collected in Cameroon and the DRC.^26^ In the present study, we investigated, for the first time, the relationship between price and quality in these 711 medicine samples, considering factors such as the types of medicine vendors/health facilities, countries of collection, medicine categories (e.g., originator or generic medicine), countries of manufacturing, and individual manufacturers. To the best of our knowledge, this is the most comprehensive investigation of the relationship between medicine price and medicine quality in LMICs published so far.

## MATERIALS AND METHODS

### Purchase of medicine samples.

The medicine samples were purchased in the course of two medicine quality studies,^15^^,^^24^ conducted according to the Guidelines on the Conduct of Surveys of the Quality of Medicines published by the WHO^27^ and the Medicine Quality Assessment Reporting Guidelines.^28^ Twelve different types of medicines were included in Cameroon and the DRC and 13 types in Nigeria. Selection of the medicine types was based on the essential medicines list of the respective countries and on information by local partners about the local importance of the medicines. This resulted in a similar but not identical selection in the three countries (Table 1). Each medicine was collected in a preferred strength if available, otherwise in an available strength, but only in adult dosages (Table 1).

**Table 1:** Medicines investigated in this study

API	Dosage Form	Strength (mg)	No. of Samples Collected in			Total	MSH Reference Price (US cents/unit)
			Cameroon	DRC	Nigeria		
Amoxicillin	Tablet/Capsule	250	0	17	0	17	1.60
		500	23	13	0	36	3.00
Amoxicillin/Clavulanic Acid	Tablet	500/125	6	4	0	10	16.41
		875/125	6	2	0	8	15.75
Atenolol	Tablet	50	0	1	14	15	1.07
		100	0	5	0	5	1.78
Ceftriaxone	Powder for Injection	1,000	0	0	22	22	39.80
Cefuroxime Axetil	Tablet	500	0	0	24	24	39.11
Chloroquine	Tablet	250	0	0	16	16	1.37
Ciprofloxacin	Tablet	500	21	29	25	75	3.73
Co-trimoxazole	Tablet	400/80	21	30	17	68	1.20
		800/160	1	0	0	1	2.41
Dexamethasone	Tablet	0.5	0	0	18	18	0.70
Doxycycline	Tablet/Capsule	100	20	25	0	45	1.33
Fluconazole	Tablet/Capsule	150	0	0	22	22	6.92
Furosemide	Tablet	40	16	23	14	53	0.61
Glibenclamide	Tablet	5	18	0	18	36	0.57
Hydrochlorothiazide	Tablet	25	8	6	15	29	0.43
		50	10	0	0	10	0.49
Metformin	Tablet	500	18	6	22	46	1.50
		850	1	5	0	6	2.00
		1,000	0	1	0	1	11.88
Metronidazole	Tablet	200	0	9	22	31	0.61
		250	13	20	0	33	0.61
		400	0	0	3	3	1.18
		500	11	0	0	11	1.18
Penicillin V	Tablet	250	6	26	0	32	1.76
		500	8	0	0	8	2.43
Salbutamol	Tablet	2	8	0	0	8	0.25
		4	6	16	0	22	0.32
Total	–	–	221	238	252	711	–

API = active pharmaceutical ingredient; DRC = Democratic Republic of the Congo; MSH = Management Sciences for Health. Several of the APIs were contained in the investigated medicines in the form of their salts (e.g., ciprofloxacin hydrochloride). These are specified in the publications on the medicine quality analysis.^15^^,^^24^ Reference prices were obtained from the MSH International Medical Products Price Guide.^30^^,^^31^ Metronidazole 200-mg and 250-mg tablets and 400-mg and 500-mg tablets are listed in that guide with the same price. For easier readability, prices are shown in US cents rather than US dollars.

In Cameroon and the DRC, medicines were purchased on a retail level, including government health facilities, church health facilities, private pharmacies, and informal vendors (total: 34 outlets in Cameroon and 26 in the DRC).^15^^,^^26^ Samples were collected in 2017/2018 from six regions of Cameroon and four provinces of the DRC.

In Nigeria, medicines were purchased on a wholesale level, either from licensed pharmaceutical manufacturers or wholesalers (hereafter referred to as *licensed vendors*) or from vendors in pharmaceutical markets with unclear licensing status (hereafter referred to as *markets*).^24^^,^^29^ Samples were collected in 2021/2022 in the states of Enugu and Anambra from a total of 62 commercial sources.

Selection of the sampling sites and the procedure of sample collection are described in detail in the publications of the previous studies.^15^^,^^24^^,^^26^ In short, sampling sites were chosen randomly in Cameroon and the DRC and purposefully in Nigeria. For the first study, carried out in Cameroon and the DRC, no prior sample size calculation was done, and 506 samples were collected. That study provided data on the rate of SF medicines detectable with the analytical methods used, and these data were used in the subsequent study in Nigeria to calculate the sample size required to find a significant difference between formal and informal sectors with 95% confidence and a power of 80% (i.e., 260 samples). In public and church health facilities in Cameroon and the DRC, local investigators identified themselves and explained the purpose of the study, whereas in private pharmacies and illegal market vendors of medicines, samples were collected using a mystery shopper approach. In Nigeria, local investigators purchased the samples in both the formal and informal sectors without mentioning the intended medicine quality testing.

Of each sample, if possible, 100 tablets/capsules or 20 vials were purchased. If only an amount smaller than 100 tablets/capsules or 20 vials was available, this amount was collected, but not less than 30 tablets/capsules or five vials per sample. Samples were purchased in their original containers if possible. Otherwise, they were collected in light- and air-tight screw-cap plastic containers carried by the investigators. In Nigeria, if the respective medicine was only sold in packages larger than 100 tablets/capsules or 20 vials, the entire package was purchased, resulting in 57 of the 252 Nigerian samples comprising ≥200 units. In contrast, in Cameroon and the DRC, only three of 459 samples comprised ≥200 units.

### Analysis of medicine quality.

Samples were investigated in the pharmaceutical institute of the University of Tübingen, Germany, for assay (quantity of the API[s]) and for dissolution, using high performance liquid chromatography according to the USP, as described in previous publications.^15^^,^^24^ Dissolution testing was not applicable for injectable formulations such as ceftriaxone powder for injection. Furthermore, it was not carried out for fluconazole capsules, as no dissolution testing method is specified in the USP, and not for eight further samples containing no or extremely low amounts of the APIs.^24^ Therefore, dissolution values were available for 674 of the 711 samples included in this study. Uniformity of dosage units had been determined for several of the medicine types in the study in Cameroon and the DRC,^15^ but not in the study in Nigeria,^24^ and was therefore not included in the present evaluation.

For all samples from Cameroon and the DRC, after completion of the chemical analysis an authenticity inquiry was carried out, asking the stated manufacturers and distributors whether these medicines represented genuine products of their company.^25^ In seven cases, the manufacturers stated that these products were falsified (see Results). This authenticity inquiry increased the number of falsified samples identified in Cameroon and the DRC from three (found by chemical analysis) to 10 samples. In contrast, for the medicine samples collected in Nigeria, only chemical analysis was carried out but no authenticity inquiry. The reported rate of falsified samples is therefore not directly comparable between the two studies and may represent an underestimation for Nigeria.

### Definitions of medicine quality.

The current definitions for “substandard” and “falsified” medicines by the WHO were used.^1^ According to the results of quality analyses and authenticity inquiries, samples were classified into the following quality categories defined in previous publications^2^^,^^16^^,^^25^: 1) “in specification”; 2) “moderate deviation” (i.e., deviations from USP assay specifications by not more than 20% of the declared API amount and/or dissolution values below USP specifications but not lower than Q minus 25% on average); 3) “extreme deviation” (i.e., containing <80% but ≥50% of the declared API amount and/or average dissolution values lower than Q minus 25%); 4) “<50% of the declared API amount” (i.e., API content less than 50% of the declared amount, possibly indicating fraudulent intent in their manufacturing); and 5) “Falsified” (see Results).^1^^,^^2^^,^^25^

### Included and excluded samples.

**Figure 1.**
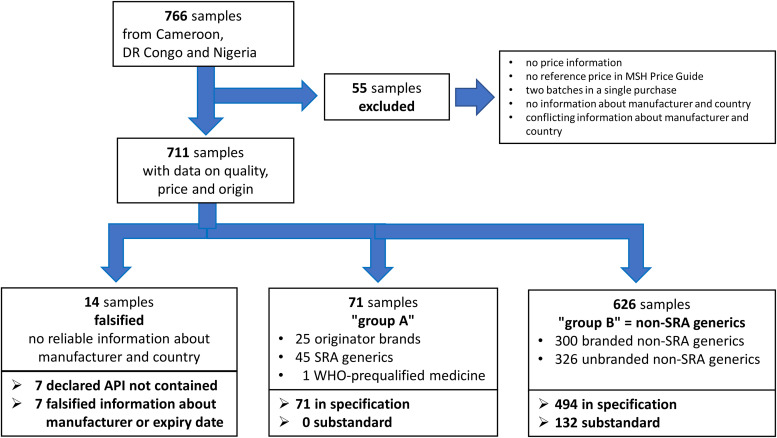
Flowchart showing the evaluation of medicine price and quality data. API = active pharmaceutical ingredient; DRC = Democratic Republic of the Congo; MSH = Management Sciences for Health; SRA = stringent regulatory authority.

Five hundred and six medicine samples had been investigated for their quality in Cameroon and the DRC^15^ and 260 in Nigeria,^24^ resulting in a total of 766 samples. Of these, 711 could be included into the present evaluation of prices and quality of medicines (Figure 1). The other 55 samples had to be excluded as no information on the individual prices of these samples had been recorded^26^ or no reference price was given in the Management Sciences for Health (MSH) price guide^30^^,^^31^ for their specific strength. Seven purchased samples had been found to contain blisters of two different batches, resulting in a single price but two quality results for each of these seven purchases.^15^^,^^24^ The two quality results were found to be similar in each of the seven cases. Only the quality result for the batch representing the larger number of units in the respective purchase was included in the present study. One sample consisting of tablets sold in a plastic bag by an informal vendor in Cameroon was excluded because no information on brand name and manufacturer was available. For one further sample (glibenclamide 5-mg tablets by “ZLF Pharma”), contradictory information on the country of manufacturing was found, and it was therefore excluded (see Results).

### Calculation of price ratios to the international reference price.

The WHO and Health Action International (HAI) have developed a standardized methodology for the comparative investigation of medicine prices, especially for LMICs.^32^ This methodology was used in the present study. Prices and quantities of purchased medicines were recorded by local investigators on standardized forms. Medicine prices were converted from national currency to US Dollars (USD) using the exchange rates given by the European Commission^33^: Cameroon, 1,000 West African CFA Franc (XOF) = 1.81933 USD (January 2018); DRC, 1,000 Congolese Franc (CDF) = 0.63244 USD (January 2018); Nigeria, first sampling period, 1,000 Naira (NGN) = 2.43841 USD (August 2021); and Nigeria, second sampling period, 1,000 Naira (NGN) = 2.41039 USD (June 2022). For each sample, the medicine price per dosage unit (tablet, capsule, or vial) was calculated. Subsequently the ratio of the price per unit to the international reference price given in the MSH International Medical Products Price Guide^30^^,^^31^ for the same medicine and strength (see Table 1) was calculated. From the medicine price ratios of all samples for a given type of medicine or type of collection site in a country, the median price ratio (MPR) was calculated as suggested by WHO/HAI.^32^ In the figures in the Results section, the ranges of price ratios are depicted as box plots, showing minimum, 25th percentile, median, 75th percentile, and maximum values.

Including all individual samples, the overall MPR in Cameroon was 5.69, in the DRC 2.17, and in Nigeria 1.41. These different overall MPRs reflect the sample collection at different levels (retail level in Cameroon and DRC, wholesale level in Nigeria), as well as the different economic status of Cameroon and the DRC^34^ and other factors. Price ratios are therefore not directly comparable between the three countries, and data were analyzed separately for each country wherever possible. When price ratios of different countries were combined, a correction for the different price levels in each country was attempted by the calculation of a “country-normalized price ratio,” dividing each individual medicine price ratio by the above-mentioned overall MPR of the respective country and thereby setting the overall country-normalized MPR for each country to 1.00.

### Classification of medicine categories.

The above-mentioned standardized methodology by the WHO/HAI^32^ recommends separately assessing the prices of “originator brands” and of generic products. The WHO/HAI define an originator brand as the product of an API that was first authorized worldwide for marketing (usually as a patented product) on the basis of the manufacturer’s documentation of its efficacy, safety, and quality.^32^ In some cases, the original manufacturer also licenses the production of the originator brand to other manufacturers. Generic medicines, on the other hand, are defined as products intended to be interchangeable with the originator brand products, usually marketed after the expiry of a patent or other exclusivity rights and usually manufactured without requiring a license from the originator manufacturer.^32^

Large international agencies such as the Global Fund to Fight AIDS, Tuberculosis and Malaria^35^ and the Global Drug Facility^36^ usually demand that generic medicines procured by them are either prequalified by the WHO Medicine Prequalification Program^37^^,^^38^ or approved by a stringent regulatory authority (SRA).^39^^,^^40^ The SRAs include the national medicine regulatory agencies of the member states of the European Community and of the United States, Canada, United Kingdom, Iceland, Liechtenstein, Norway, Switzerland, Japan, and Australia. Major international purchasers such as the Global Fund and Global Drug Facility consider that medicines with a marketing authorization issued by an SRA or with a prequalification by the WHO Medicine Prequalification Program can be relied upon and do not need reassessment to be eligible for procurement.^40^ The WHO has started to replace the concept of SRAs with the new concept of “WHO-listed authorities (WLAs),”^40^ but at the time of writing (May 2024), the WLAs still comprised authorities from only three countries.^41^ In this study, all authentic generic medicines stated to be manufactured in a country with an SRA were classified as “SRA generics,” without confirming whether indeed a marketing authorization had been issued by the respective SRA for that medicine. Authentic generic medicines stated to be manufactured in other countries were classified as “non-SRA generics.”

Generic medicines are marketed either under the international nonproprietary names of the respective APIs (“unbranded generics”) or under a brand name chosen by the marketing authorization holder (“branded generics”). Very different pricing and marketing policies for branded and unbranded generics produced by the same manufacturer have been reported (e.g., from India^18^ and Indonesia).^19^ However, information on price differences between branded and unbranded generics (from the same or different manufactures) in other countries is lacking,^42^ and was therefore investigated in the present study, focusing on the non-SRA generics that represent the major part of the samples in this study.

For falsified medicines, no reliable information was available about the name and location of their manufacturer; therefore, they could not be placed into any of the categories mentioned above. They were placed into a separate category in this study (Figure 1), as had been done by Bate at al.^12^

Based on the above-mentioned criteria, the medicines in the present study were therefore classified in the initial analyses into the following six categories: originator brands, SRA generics, WHO-prequalified medicines, branded non-SRA generics, unbranded non-SRA generics, and falsified medicines. Based on observed similarities between these individual categories and on the fact that originator brands are usually first approved by an SRA, originator brands and SRA generics (and the single WHO-prequalified medicine) were subsequently combined into “group A” and branded and unbranded non-SRA generics into “group B” (Figure 1). In this study, substandard medicines were exclusively found within group B (i.e., within the non-SRA generics); therefore, many of the analyses were carried out for this group alone.

## STATISTICAL ANALYSES

Statistical analyses were done using SPSS for Windows release 29 (IBM Corp., Armonk NY). The relationship between medicine categories and medicine quality (dichotomized) was investigated by a χ^2^ test. For comparison of prices between medicine categories, a one-factorial analysis of variance (ANOVA) with pairwise comparisons using Tukey’s (B) test was carried out. For this analysis, the prices were logarithmically transformed (basis 10). The descriptive analyses of medicine prices used medians and quartiles on the original scale. Furthermore, the Mann-Whitney *U* test was used to investigate differences between price ratios of group A and group B explained above. Binary logistic regression was applied to investigate the association between the (logarithmically transformed) country-normalized price ratio and the quality of medicines (dichotomized). Spearman correlations were calculated for the correlation of the exact quantitative results of assay and dissolution testing with medicine prices. For statistical testing of differences between proportions, MedCalc® (MedCalc Software Ltd, Ostend, Belgium) was used.^43^ The level of significance was 0.05 (two-sided) in all statistical tests. As the primary analysis was descriptive, no adjustment for multiple testing was applied except for Tukey’s (B) test.

## RESULTS

### Number of samples from different types of collection sites and from different medicine categories.

In Cameroon and the DRC, medicines were purchased on the retail level, and in Nigeria on the wholesale level (see Materials and Methods). Table 2 shows the number of samples, as well as the number of different brands/products purchased from different types of collection sites and their classification into different medicine categories (see Materials and Methods). The smaller number of samples collected from government health facilities in Cameroon and the DRC compared with other types of collection sites reflects the lower availability of medicines in the government facilities.^26^ In contrast, both the informal vendors in Cameroon and the DRC and the “open drug markets” in Nigeria (which are not simple roadside market stalls, but shops with professional appearance)^24^ were found to be well stocked with different medicines. The total number of medicine samples collected was similar in each of the three countries.

**Table 2:** Numbers of samples with numbers of different brands/products (in parentheses) from different types of collection sites and from different medicine categories

	Cameroon	DRC	Nigeria	Total
Type of Collection Site				
Government Health Facilities	35 (28)	29 (25)	n.a.	64 (53)
Church Health Facilities	62 (40)	65 (45)	n.a.	127 (83)
Private Pharmacies	60 (34)	88 (67)	n.a.	148 (100)
Informal Vendors	64 (46)	56 (45)	n.a.	120 (91)
Licensed Vendors	n.a.	n.a.	107 (79)	107 (79)
Markets (licensing unclear)	n.a.	n.a.	145 (102)	145 (102)
Total	221 (119)	238 (126)	252 (159)	711 (399)
Medicine Category				
Originator Brand	14 (5)	9 (2)	2 (2)	25 (8)
SRA Generic	36 (19)	8 (7)	1 (2)	45 (27)
WHO-Prequalified Product	1 (1)	0 (0)	0 (0)	1 (1)
Branded Non-SRA Generic	44 (27)	101 (58)	155 (97)	300 (182)
Unbranded Non-SRA Generic	118 (63)	118 (58)	90 (55)	326 (172)
Falsified Medicine	8 (5)	2 (2)	4 (4)	14 (11)
Total	221 (119)	238 (126)	252 (159)	711 (399)

DRC = Democratic Republic of the Congo; INN = international nonproprietary name; n.a. = not applicable (different types of collection sites were investigated in the different countries); SRA = stringent regulatory authority. See Materials and Methods for explanation of medicine categories. Medicines with different brand or INN names, with different strengths, from different manufacturers, or from different marketing authorization holders were considered as different brands/products. (The WHO has started to replace the concept of SRAs with the new concept of “WHO-listed authorities [WLAs],”^40^ but at the time of writing [May 2024], the list of WLAs still comprised authorities from only three countries.^41^)

Only relatively few of the collected samples represented originator brands or generic medicines from a country with an SRA (i.e., SRA generics; see Materials and Methods), and only a single one represented a WHO-prequalified product. Most of the samples were non-SRA generics, either branded or unbranded.

**Figure 2.**
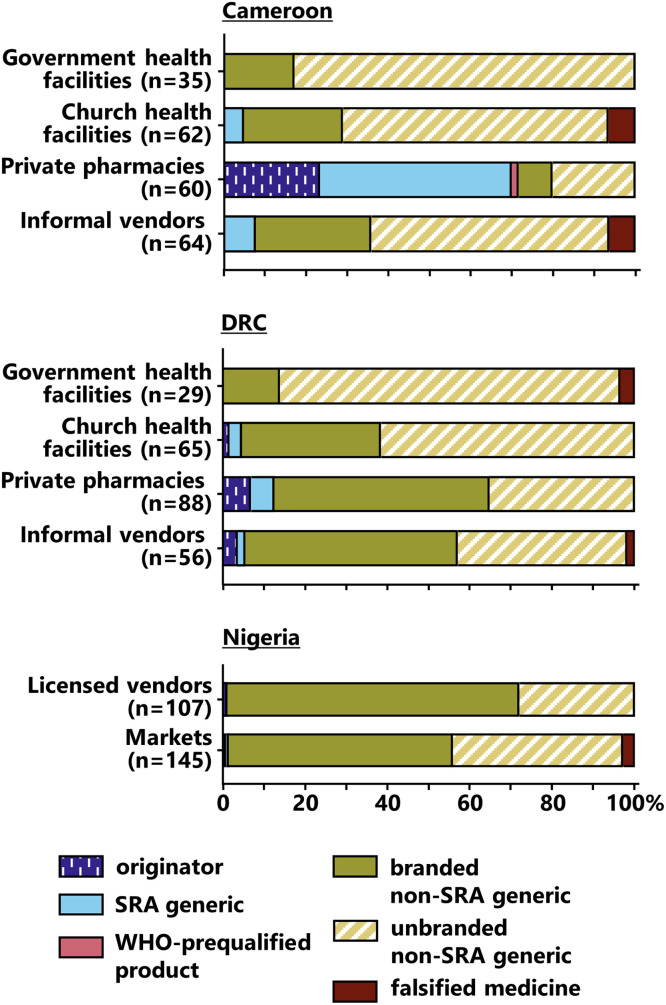
Percentage of samples in different medicine categories found in different types of collection sites. The precise numbers of samples in different categories are shown in Supplemental Table 1. DRC = Democratic Republic of the Congo; SRA = stringent regulatory authority.

Figure 2 shows that most of the originator medicines and SRA generics were found in Cameroon, especially in private pharmacies. In contrast, in Nigeria nearly all of the samples were non-SRA generics; in that country, wholesale sources for medicine procurement accessible to a faith-based medicine supply organization had been investigated.^24^

### Falsified medicines.

A total of 14 medicine samples, listed in Table 3, were identified as falsified. They were found in both the formal and informal sectors (Figure 2). All aforementioned categories of medicines were affected by falsification: Of 26 samples labeled as originator brands, one (4%) was found to be falsified. Of 46 products labeled as SRA generics, one (2%) was falsified, for branded non-SRA generics three of 303 (1%) and for unbranded non-SRA generics nine of 335 samples (3%).

**Table 3:** Fourteen medicine samples identified as falsified

Stated Product Name	Stated API	Stated Manufacturer	Stated Country of Manufacturing	Country Of Collection	Type Of Collection Site	Result Of Chemical Analysis^15^^,^^24^	Result Of Authenticity Inquiry and Packaging Analysis
Augmentin	Amoxicillin/Clavulanic Acid	SmithKline Beecham Pharmaceuticals*	United Kingdom	Cameroon	Informal Vendor	Stated API Not Contained	Not Authentic^15^
Penicillin-V Tablets	Penicillin V	Oxford Pharma	Belgium	Cameroon	Informal Vendor	Stated API Not Contained^†^	Stated Manufacturer Does Not Exist^15^
Metronyl	Metronidazole	MAC’S Pharmaceuticals Ltd.*	Kenya	DRC	Informal Vendor	Stated API Not Contained^‡^	No Response Received^15^
Rotrim	Co-trimoxazole	ROTAC MEDICAL LABORATORY	Nigeria	Nigeria	Market	Stated API Not Contained	Stated Manufacturer Does Not Exist^24^
Weltrim	Co-trimoxazole	WELTEC HEALTHCARE LTD	Nigeria	Nigeria	Market	Stated API Not Contained^†^	Stated Manufacturer Does Not Exist^24^
C Cotrim - 480	Co-trimoxazole	CITICARE LABORATORY LTD	Nigeria	Nigeria	Market	Stated API Not Contained	Stated Manufacturer Does Not Exist^24^
Chloro	Chloroquine	LEOBEN HEALTHCARE	Nigeria	Nigeria	Market	Stated API Not Contained	Stated Manufacturer Does Not Exist^24^
Co-Trimoxazole	Co-trimoxazole	Sprukfield*	Togo	Cameroon	Informal Vendor	Compliant with Specifications	Not Authentic^25^
Amoxicillin + Clavulanic acid BP	Amoxicillin/Clavulanic Acid	Medopharm Pvt. Ltd.*	India	Cameroon	Informal Vendor	Compliant with Specifications	Not Authentic^25^
Metronidazole Tablets BP	Metronidazole	Medopharm Pvt. Ltd.*	India	DRC	Government Health	Compliant with Specifications	Manipulated Expiry Date^25^
Furosemide 40 mg BP	Furosemide	Micro Laboratories Ltd.*	India	Cameroon	Church Health Facility	Compliant with Specifications	Manipulated Expiry Date^25^
Furosemide 40 mg BP	Furosemide	Micro Laboratories Ltd.*	India	Cameroon	Church Health Facility	Compliant with Specifications	Manipulated Expiry Date^25^
Furosemide 40 mg BP	Furosemide	Micro Laboratories Ltd.*	India	Cameroon	Church Health Facility	Compliant with Specifications	Manipulated Expiry Date^25^
Furosemide 40 mg BP	Furosemide	Micro Laboratories Ltd.*	India	Cameroon	Church Health Facility	Compliant with Specifications	Manipulated Expiry Date^25^

API = active pharmaceutical ingredient; DRC = Democratic Republic of the Congo.

* Note that the stated manufacturer usually is a victim rather than the perpetrator of falsifications of its products.

^†^ Contained paracetamol instead of the stated API(s).

^‡^ Contained metronidazole benzoate instead of the stated API.^15^^,^^24^

Of the 14 falsified medicines, seven did not contain the stated API(s) (Table 3). Notably, the other seven were found to comply with pharmacopeial specifications for assay and dissolution; they were identified as falsified by authenticity inquiries to their stated manufacturers^25^ and/or by packaging analysis.^15^^,^^24^ In the cases of two of these falsifications, tablets with the correct ingredients had been produced (or otherwise obtained) but were packaged imitating the packaging of two authentic manufacturers of generic medicines (located in Togo and India, respectively). Possibly, this was done to take advantage of existing marketing authorizations for the products by these manufacturers. For the other five products, their expiry dates had been manipulated (i.e., had been fraudulently extended beyond the genuine expiry date given by the manufacturer). Although it is theoretically possible that these seven falsified products, which complied with specifications, were still therapeutically effective, they could not be considered safe.

### Medicine prices and medicine quality in different types of collection sites.

**Figure 3.**
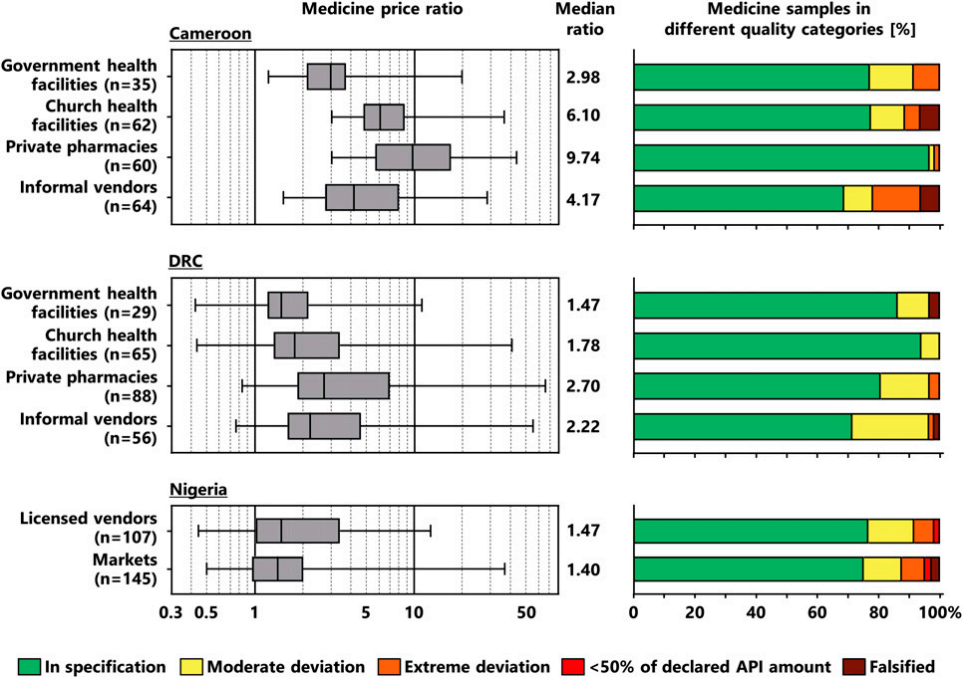
Medicine prices and medicine quality in different types of collection sites. See Materials and Methods for calculation of price ratios to the international reference price published by Management Sciences for Health^30^ and for the definition of the quality categories. Box plots for the price ratio show minimum, 25th percentile, median, 75th percentile, and maximum values. Falsified medicines were detected in Cameroon and the DRC using both chemical analysis and an authenticity inquiry,^15^^,^^25^ but in Nigeria using chemical analysis alone^24^ (see Materials and Methods). The depicted rates of falsified medicines may therefore represent an underestimate for Nigeria. The numerical values for medicine samples in different quality categories are shown in Supplemental Table 2. API = active pharmaceutical ingredient; DRC = Democratic Republic of the Congo.

Figure 3 shows the medicine prices observed in different types of collection sites. As suggested by the WHO/HAI,^32^ medicine prices were expressed as price ratio to the international reference price published by Management Sciences for Health^30^^,^^31^ for medicines of the respective composition and strength (see Materials and Methods). Similar to previous observations,^11^^,^^12^^,^^19^ in each type of collection site, price ratios varied over quite a wide range. The MPR was higher in private pharmacies than in other types of collection sites, both in Cameroon and the DRC (Figure 3). In each type of collection site, prices in Cameroon were higher than prices in the DRC, possibly reflecting the higher economic status of Cameroon.^34^ In Nigeria, medicines were purchased on the wholesale level, resulting in lower medicine prices compared with prices in Cameroon and the DRC. The price ratios are therefore not directly comparable between the three countries (see Materials and Methods).

As explained in Materials and Methods, the results of the medicine quality investigations were used to place the samples into five quality categories, depicted on the right side of Figure 3. The highest percentages of SF samples were observed in informal vendors (Cameroon, DRC) and markets with unclear licensing status (Nigeria). However, SF medicines were also observed in the formal sector. In Cameroon, the percentage of SF samples was lowest in private pharmacies (Figure 3), where the medicine prices (Figure 3) and the percentages of originator brands and SRA generics (Figure 2) were highest.

In Nigeria, all four detected falsified medicine samples had been found in markets with unclear licensing status.^24^ In Cameroon and the DRC, the three falsified samples that did not contain the stated API (Table 3)^15^ and the two falsified samples that showed the correct API content but had not been produced by the stated manufacturer (Table 3)^25^ were found in informal vendors. However, of the five samples with fraudulently manipulated expiry dates, four (all representing the same medicine type and brand) were found in church health facilities in Cameroon and one in a government health facility in the DRC (Table 3). In this study, no falsified medicines were found in the private pharmacies investigated in Cameroon and the DRC.

### Prices and quality in different categories of medicines.

**Figure 4.**
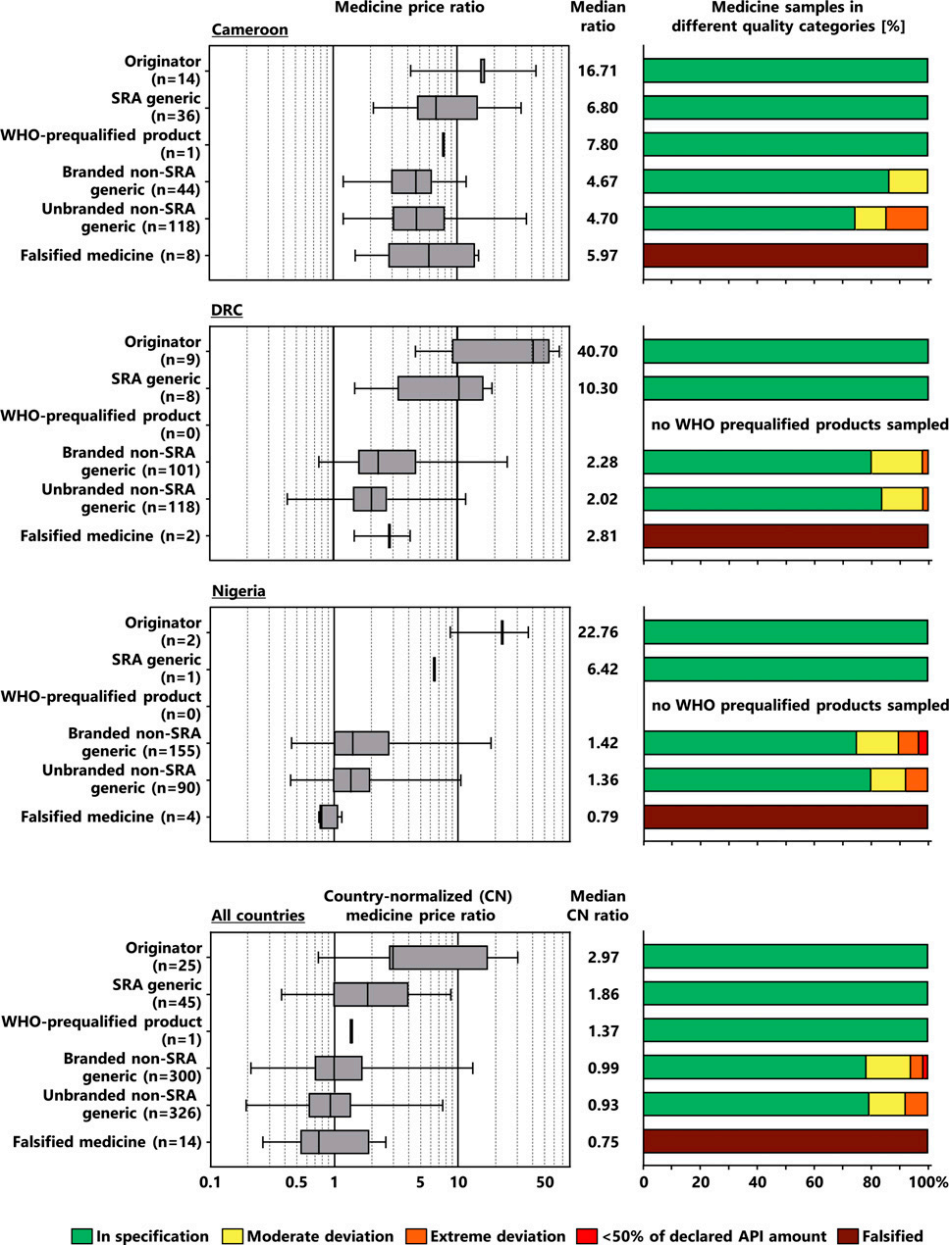
Medicine prices and medicine quality in different categories of medicines. In the panels for “All countries,” the data from the three countries are combined. For this purpose, country-normalized price ratios were calculated, setting the overall price ratio for each country to 1.00 (see Materials and Methods). For some of the medicine categories, only one or very few samples were found, and no generalization of the results was attempted in these cases. Box plots for the price ratio show minimum, 25th percentile, median, 75th percentile, and maximum values. The numerical values for medicine samples in different quality categories are shown in Supplemental Table 2. API = active pharmaceutical ingredient; DRC = Democratic Republic of the Congo; SRA = stringent regulatory authority.

Figure 4 shows the prices and quality for the different medicine categories defined in Materials and Methods. In each country, branded non-SRA generics and unbranded non-SRA generics were found to have nearly the same MPR. The SRA generics were overall approximately twice as expensive and originator brands three times as expensive as the non-SRA generics. The order of the prices of these categories of medicines was identical in each of the three countries. However, in the DRC the prices of originators and SRA generics were much higher than those of the non-SRA generics, but this observation is based only on a small number of originator and SRA generic samples. As mentioned above, only a single WHO-prequalified medicine was found in the entire study.

An ANOVA with pairwise comparison using Tukey’s (B) test (see Materials and Methods) confirmed a highly significant relationship between medicine categories and medicine prices (*P <*0.001). Prices were lowest and not significantly different between branded and unbranded non-SRA generics, but were significantly higher for SRA generics and originator brands. Moreover, the prices of originator brands were significantly higher than those of SRA generics (Supplemental Table 3).

Highly significant differences (*P <*0.001, χ^2^ test) were observed for the quality of medicines between the different categories. Without exception, all originator brands and SRA generics (as well as the single WHO-prequalified medicine) were found to comply with pharmacopeial specifications for assay and dissolution. Substandard samples were only found in non-SRA generics, with no significant differences in the percentages between branded and unbranded non-SRA generics (21.7% and 20.6%, respectively; *P* = 0.733, χ^2^ test).

As mentioned above, falsified medicines were placed in a separate category. Their prices were somewhat higher than those of non-SRA generics in Cameroon and the DRC and somewhat lower in Nigeria. The falsified medicine labeled as an SRA generic (“Penicillin V tablets”; Table 3) was similar in price to other SRA generics, but the falsified medicine labeled as an originator brand (“Augmentin tablets”) was conspicuously cheap, showing a price ratio to the MSH reference price 11 times lower than the MPRs of the other originator medicines in this study. However, the sample numbers of falsified medicines were low and did not allow generalized conclusions.

The above-mentioned observations suggest that the five categories of nonfalsified medicines shown in Figure 4 may be combined into two principal groups, as shown in Table 4. The first group (hereafter called group A) comprised originator brands (which are usually first approved by an SRA), SRA generics, and the single WHO-prequalified medicine (total 71 samples). All of the samples in this group were compliant with pharmacopeial specifications for assay and dissolution. The other group (hereafter called group B) comprised the 626 samples of generic medicines from countries without stringent regulatory authorities (i.e., non-SRA generics, branded or unbranded). In this group, 132 samples (21.1%) were substandard (95% CI: 17.6–25.0%). For group A, the median country-normalized price ratio was 2.69, and for group B it was 0.97. Therefore, within the samples investigated in this study, medicines of group A were approximately 2.8 times more expensive than the non-SRA generics (group B). The differences between groups A and B in the percentage of substandard samples and in the median country-normalized price ratios were statistically significant (*P <*0.0001 for both comparisons). As shown in Table 4, for the DRC and for Nigeria, the numbers of medicine samples from group A were low (17 and 3, respectively), and the quality differences to group B were not statistically significant for these countries alone.

**Table 4:** Comparison of medicine prices and medicine quality between A) medicines representing originator brands, SRA generics, or a WHO-prequalified medicine and B) generic medicines from countries without SRAs*

Groups of Medicines	Median Price Ratio			Median CN Price Ratio	% Substandard Samples*			
	Cameroon	DRC	Nigeria	All Countries	Cameroon	DRC	Nigeria	All Countries
Group A Medicines Representing Originator Brands, SRA Generics, or a WHO-Prequalified Medicine	12.17 (*n =* 51)	14.21 (*n =* 17)	8.67 (*n =* 3)	2.69 (*n =* 71)	0.0% (*n =* 51)	0.0% (*n =* 17)	0.0% (*n =* 3)	0.0% (*n =* 71)
Group B Non-SRA Generics (branded or unbranded)	4.70 (*n =* 162)	2.11 (*n =* 219)	1.41 (*n =* 245)	0.97 (*n =* 626)	22.2% (*n =* 162)	17.8% (*n =* 219)	23.3% (*n =* 245)	21.1% (*n =* 626)
*P*-Value for Difference between Groups A and B	*<*0.001^†^	*<*0.001^†^	0.005^†^	*<*0.0001^†^	0.0002	0.057	0.34	*<*0.0001

CN price ratio = country-normalized price ratio (see Materials and Methods); DRC = Democratic Republic of the Congo; SRA = stringent regulatory authority.

* The 14 falsified samples were not included in this calculation, as they cannot be placed into any of the two categories compared here. The single WHO-prequalified medicine found in this study, manufactured in India, was included under group A.

^†^ Asymptotic significance, 2-tailed (Mann-Whitney *U* test).

### Non-SRA generic medicines with misleading labeling information, suggesting origin in an SRA country.

**Figure 5.**
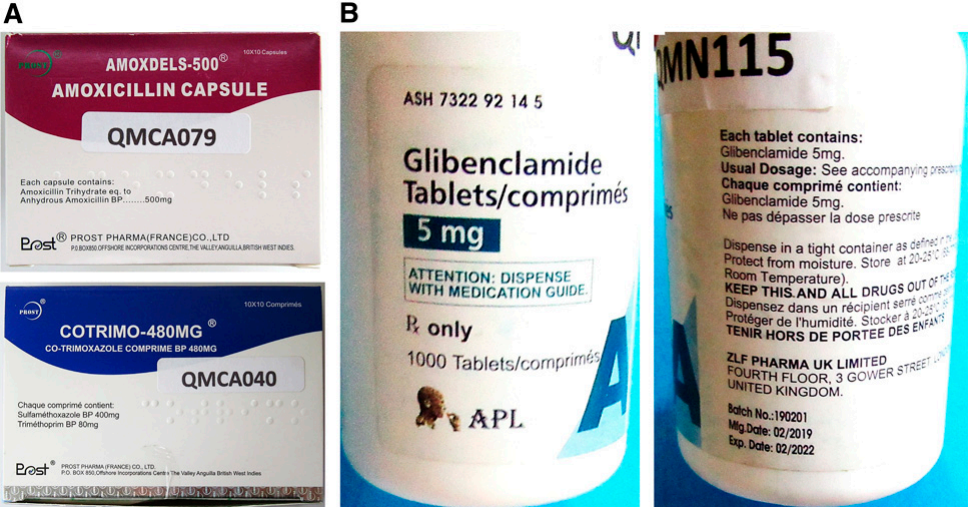
Samples of non-SRA generic medicines with misleading labeling information, suggesting their origin in an SRA country. (**A**) The labels of the two samples on the left state company of origin as “Prost Pharma (France) Co., Ltd.” and address as “P.O. Box 850, Offshore Incorporations Center, The Valley, Anguilla, British West Indies.” However, they are apparently manufactured in China (www.prost-pharma.com; see text). (**B**) The label of the samples on the right state company of origin as “ZLF Pharma UK Limited” and address as “Fourth Floor, 3 Gower Street, London, United Kingdom.” However, it is most likely also manufactured in China (www.zlfpharma.com; see text). SRA = stringent regulatory authority.

As mentioned in Materials and Methods, major international medicine purchasers such as the Global Fund^35^ and the Global Drug Facility^36^ consider that medicines with a marketing authorization issued by an SRA can be relied upon and do not need reassessment to be eligible for procurement.^40^ Our findings described above are consistent with this notion. However, some manufacturers of non-SRA generics may try to take advantage of the good reputation of SRA generics by displaying misleading information on their labels. Possible examples are three samples identified in this study, depicted in Figure 5. Two samples (Figure 5A) were collected in Cameroon and represented amoxicillin capsules and co-trimoxazole tablets. The label stated a company named “Prost Pharma (France) Co., Ltd” as origin, with a post office box address in Anguilla, British West Indies. However, an internet search showed that “Prost Pharma (France) Co., Ltd” is a Chinese manufacturer located in Hangzhou City, P.R. China (www.prost-pharma.com). Product photos displayed on the website of that manufacturer were identical to those in Figure 5A. In the present study, these samples were therefore considered to be manufactured in China. Both samples complied with pharmacopeial specifications for assay and dissolution.

One further sample (Figure 5B) represented glibenclamide 5-mg tablets collected in Nigeria (stated marketing authorization holder: “APL”). The label stated as origin “ZLF Pharma UK Limited,” with a street address in London, United Kingdom. In the British government’s register of companies,^44^ this company is listed not as a pharmaceutical manufacturer but as a retail seller of medical goods; the director is stated to reside in China. An internet search for the company name led to “ZLF pharmaceutical limited” (*sic!*) (www.zlfpharma.com), a pharmaceutical manufacturer in Wuxi city, P.R. China, which lists glibenclamide 5-mg tablets among its products. However, no photos of the products were shown on the manufacturer’s website; therefore, there is no definite proof of the origin of this medicine, and it was excluded from data analysis for this study (see Materials and Methods). This sample failed pharmacopeial specifications for dissolution.

Therefore, when the quality of medicines from different countries is compared, care must be taken to avoid mistakes arising from misleading information on the label.

### Prices and quality of non-SRA generics from different countries of manufacturing.

**Figure 6.**
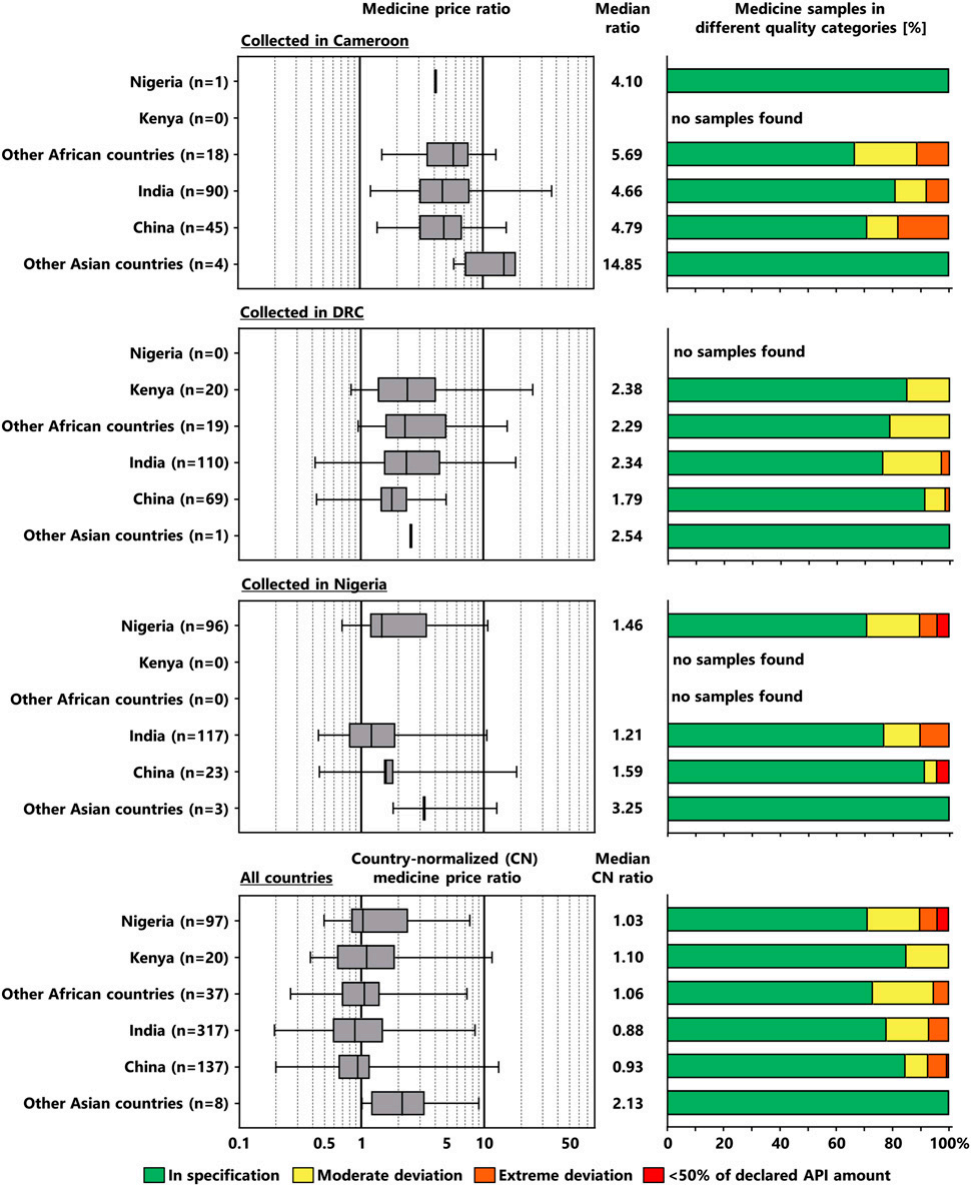
Prices and quality of generic medicines manufactured in different non-SRA countries. Note that for some countries of manufacturing, only very few samples were found in individual countries of sample collection. Falsified medicines were not included in this evaluation, as no reliable information is available on their country of manufacturing. Also, the single WHO-prequalified medicine found in this study (manufactured in India) was not included. Box plots for the price ratio show minimum, 25th percentile, median, 75th percentile, and maximum values. The numerical values for medicine samples in different quality categories are shown in Supplemental Table 2, and information on the prices and quality from all individual countries of manufacturing is given in Supplemental Table 4. DRC = Democratic Republic of the Congo; SRA = stringent regulatory authority.

Figure 6 compares prices and quality of generic medicines stated to be manufactured in those non-SRA countries that were most frequently encountered in this study. In addition, Supplemental Table 4 compares price and quality data for all countries of manufacturing encountered in this study. As obvious from Figure 6, the prices of the medicines manufactured in the different countries were similar. The overall percentages of substandard samples in medicines stated to be manufactured in Nigeria, Kenya, India, and China were 28.9%, 15.0%, 23.3%, and 15.3%, respectively.

In numbers of samples, India and China were strongly represented in all three countries of collection (Figure 6), together representing 73.7% of the investigated samples of non-SRA generics. On the other hand, 25.0% of the non-SRA generics were manufactured in African countries, with the highest percentage (40.2%) found in Nigeria, a country with a large number of local pharmaceutical manufacturers. In Nigeria no medicines manufactured in any other African country were found in this study. Inversely, in Cameroon and the DRC (combined) only a single sample manufactured in Nigeria was found, collected from an informal vendor in Cameroon. This indicates relatively little trading of pharmaceuticals between these countries.

### Correlation of medicine quality with medicine prices.

The box plots in Figure 4 depict that even within the non-SRA generics, medicine prices varied over a wide range. The median prices of substandard non-SRA generics were essentially the same as the prices of in-specification samples in Nigeria and even somewhat higher than those of in-specification samples in Cameroon and the DRC Supplemental Figure 1). A logistic regression analysis Supplemental Table 5) showed no significant correlation between medicine prices and medicine quality within all 711 medicine samples nor within the 697 nonfalsified medicine samples. Within the non-SRA generics, even a negative correlation between prices and quality was observed. A logistic regression model including medicine categories as predictor and quality as outcome did not converge, owing to the observation of “null cells” for originator medicines and SRA generics.

**Figure 7.**
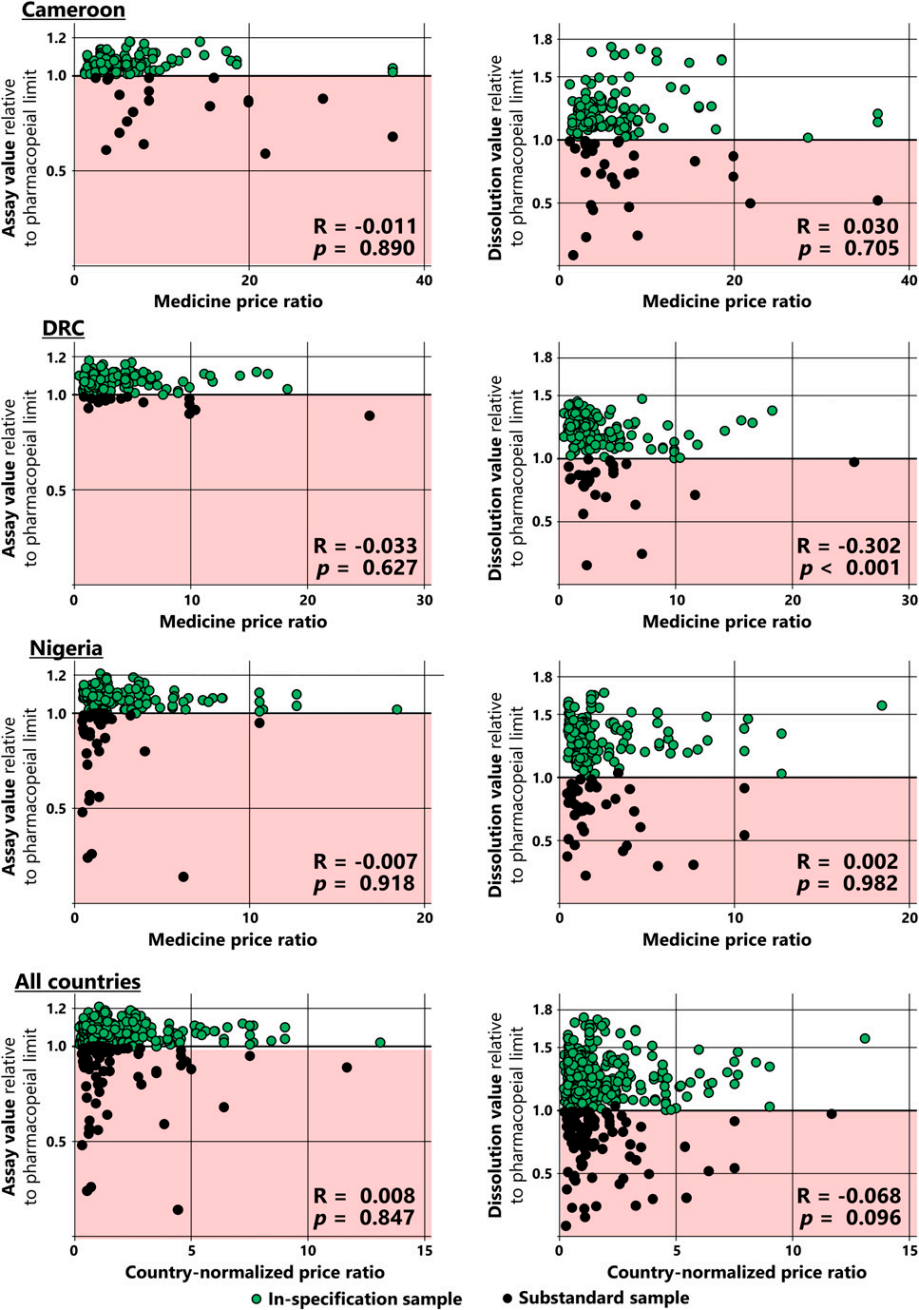
Correlation of the quantitative results of assay and dissolution testing with medicine prices. Assay and dissolution results were calculated as ratios to the lowest permissible value specified in the United States Pharmacopeia for the respective API and formulation. Thereby, all medicine samples with a value of ≥1.0 are in specification and are of satisfactory quality for the tested criterion. Medicine samples with values <1.0 are substandard, with lower values showing more severe deviations. Spearman correlation coefficients and *P*-values (2-tailed significance) were calculated. In this study, only a single sample was found that was substandard owing to an excessive (rather than insufficient) amount of the API; this sample was excluded from the data analysis presented in this figure. API = active pharmaceutical ingredient; DRC = Democratic Republic of the Congo.

In Figure 7, the exact quantitative results of assay and dissolution testing for each non-SRA generic sample are plotted against the price of that sample, and the Spearman correlation coefficients are shown. In seven of the eight panels in Figure 7, no significant correlation was observed between medicine prices and the quantitative assay and dissolution results. In one panel (DRC; dissolution values), a significant negative correlation was observed.

Therefore, although originator brands and SRA generics had significantly higher prices and significantly higher quality than non-SRA generics (see “Prices and quality in different categories of medicines”), within the non-SRA generics no evidence was found that higher prices correlated with higher quality.

### Prices and quality of non-SRA generics from different manufacturers.

The 626 samples of non-SRA generics investigated in this study had been manufactured, according to the information on the labels, by 158 manufacturers located in 16 countries. Table 5 lists the prices and quality data for the 11 manufacturers encountered most frequently (i.e., represented with at least 10 samples in this study).

**Table 5:** Prices and quality of non-SRA generic medicines from the 11 manufacturers encountered most frequently in this study

Manufacturer	Country	Sample Numbers				Median CN Price Ratio	Sample Numbers in Quality Categories				
		Total Number	Country of Collection				In Specification	Moderate Deviation	Extreme Deviation	<50% API	Total OOS
			Cameroon	DRC	Nigeria						
CSPC Zhongnuo Pharmaceutical Co. Ltd.	China	10	0	8	2	0.68	10	0	0	0	0
Medopharm Pvt. Ltd.*	India	30	9	21	0	0.68	29	1	0	0	1
CSPC Ouyi Pharmaceutical Co. Ltd.	China	19	3	16	0	0.82	18	1	0	0	1
JUHEL NIGERIA LIMITED	Nigeria	10	0	0	10	3.98	9	1	0	0	1
Strides Arcolab Limited	India	18	16	2	0	0.65	16	2	0	0	2
Mancare Pharmaceuticals Pvt. Ltd.	India	18	4	0	14	0.80	16	1	1	0	2
Astra Lifecare Pvt. Ltd.	India	11	0	11	0	1.10	9	0	2	0	2
Strides Shasun Limited	India	10	6	4	0	0.77	8	2	0	0	2
SKG-Pharma Limited	Nigeria	13	0	0	13	0.98	10	2	1	0	3
Maxtar Bio-Genics	India	15	14	0	1	1.07	10	2	3	0	5
Sinochem Jiangsu Co. Ltd.	China	14	6	8	0	0.73	8	3	3	0	6

API = active pharmaceutical ingredient; CN price ratio = country-normalized price ratio (see Materials and Methods); DRC = Democratic Republic of the Congo; OOS = out of specification; SRA = stringent regulatory authority; USP = United States Pharmacopeia. Many medicine samples were collected in health facilities, and it is unknown whether the manufacturers’ storage recommendations had been complied with from the time of manufacture until the time of sample collection. Changes in medicine quality may have occurred owing to inappropriate transport and storage conditions, and noncompliance with USP specifications is therefore not necessarily due to substandard manufacturing or packaging.

* Two further samples from Medopharm Pvt. Ltd. are listed among the falsified medicines in Table 3. These falsifications are likely to have occurred outside the responsibility of this manufacturer.

The highest number of samples (*n =* 30) was from the well-known Indian manufacturer Medopharm Pvt. Ltd. Notably, the 30 samples from this manufacturer covered 10 of the 18 types of medicines investigated in this study (Table 1). Only one genuine Medopharm sample was found to be substandard. This was a sample of metronidazole tablets collected in a church health facility in Ituri province, DRC, which showed a dissolution value of 83.4%, narrowly below the pharmacopeial limit of 85%. The rate of substandard samples among the investigated Medopharm products (one out of 30; i.e., 3.3%) was significantly lower than the rate of substandard samples among the other 596 non-SRA generics (22.0%; *P* = 0.0146). Interestingly, at the same time the median country-normalized price ratio of the 30 genuine Medopharm samples was 0.68 (i.e., 30% lower than the median ratio for all 626 non-SRA generics [0.97]). Twenty-nine of the 30 Medopharm samples had been found in government and church health facilities in Cameroon and the DRC.

Notably, one of the falsified medicines detected in this study, obtained from an informal vendor in Cameroon, fraudulently imitated a generic Medopharm product (Table 3). In a further, probably genuine sample from Medopharm, the expiry date had been fraudulently manipulated (Table 3). Both of these cases of falsification are likely to have occurred outside the responsibility of this manufacturer.

Among the other 10 manufacturers of non-SRA generics listed in Table 5, the observed rate of substandard samples ranged from as low as 0% to as high as 42.9%, including samples with extreme deviations from pharmacopeial specifications. However, the sample numbers were small, and none of the differences to the rate of substandard samples among the other non-SRA generics reached statistical significance. The observed rate of substandard samples for each manufacturer was not correlated with medicine prices.

The data in Table 5 further show that none of the 11 most frequently encountered manufacturers of non-SRA generics was found in all three countries of sample collection in this study. In fact, only two manufacturers, together representing only 11 of the 626 non-SRA generic samples, were represented with samples in all three countries. Beyond the 11 manufacturers shown in Table 5, an additional 147 manufacturers of non-SRA generics were represented with samples in this study, but only with small numbers (median: two samples per manufacturer). The quality results on the individual manufacturers are available in the supplementary data of the previous publications.^15^^,^^24^^,^^25^

### Prices and quality of non-SRA generics from manufacturers with WHO Public Inspection Reports.

As mentioned above, the WHO operates a program for the prequalification of medicines and other medical products.^37^^,^^38^ The prequalification process for finished pharmaceutical products (FPPs) includes not only the assessment of the product dossier and laboratory testing of product quality but also inspection of the specific manufacturing site of that product.^38^ A “WHO Public Inspection Report (WHOPIR)”^45^^,^^46^ is the publicly available summary of the report of the on-site inspection, indicating that the FPP manufacturing site is compliant with international standards and norms. Obviously, the findings of that report are not necessarily applicable to other manufacturing sites operated by the same company. If a WHOPIR is issued but in a following inspection the site is found to be noncompliant, the respective WHOPIR is removed from the website.^46^

Among the 158 manufacturers of non-SRA generics encountered in this study, a WHOPIR was available for 12 of them, including the above-mentioned Medopharm Pvt. Ltd. These 12 manufacturers are listed in Table 6. It must be reemphasized that the WHO prequalification for an FPP applies to a specific product manufactured at a specific site, not to all products and sites of a given manufacturer. Nevertheless, the fact that a manufacturer has applied successfully (or is applying) for WHO prequalification of one or several of its products may indicate its commitment to producing good-quality medicines. Indeed, among the 82 samples produced by the 12 manufacturers with WHOPIRs (Table 6), the rate of substandard samples (7.3%) was significantly lower than the rate among the 544 other non-SRA generics (23.2%; *P* = 0.0011). Furthermore, none of the 82 samples of these 12 manufacturers showed extreme deviations from pharmacopeial specifications, whereas 44 samples (8.1%; *P* = 0.0076) of the other 544 non-SRA generics showed extreme deviations, including some deviations by more than 50% from the declared API content. This suggests that if a medicine has been produced by a manufacturer with WHOPIRs, the likelihood of this medicine being substandard is lower than otherwise. At the time of writing (May 2024), WHOPIRs were available on the WHO website^45^^,^^46^ for 98 manufacturers of FPPs, and these are listed in Supplemental Table 6.

**Table 6:** Prices and quality of non-SRA generic medicines from manufacturers with WHOPIRs^45^^,^^46^

Manufacturer	Country	Sample Numbers				Median CN Price Ratio	Sample Numbers in Quality Categories					Significance*
		Total Number	Country of Collection				In Specification	Moderate Deviation	Extreme Deviation	<50% API	Total OOS	
			Cameroon	DRC	Nigeria							
Medopharm Pvt. Ltd.^†^	India	30	9	21	0	0.68	29	1	0	0	1	–
Strides Arcolab Limited	India	18	16	2	0	0.65	16	2	0	0	2	
Strides Shasun Limited	India	10	6	4	0	0.77	8	2	0	0	2	
Milan Laboratories (India) Pvt. Ltd	India	6	1	5	0	0.77	5	1	0	0	1	
Guilin Pharmaceutical Co. Ltd.	China	4	1	3	0	0.69	4	0	0	0	0	
Macleods Pharmaceuticals Ltd.	India	4	4	0	0	0.76	4	0	0	0	0	
North China Pharmaceutical Co. Ltd.	China	4	0	4	0	0.61	4	0	0	0	0	
Mepro Pharmaceuticals Pvt. Ltd.	India	2	1	1	0	0.78	2	0	0	0	0	
Cadila Healthcare Ltd.	India	1	0	1	0	6.55	1	0	0	0	0	
Emcure PHARMACEUTICALS LTD.	India	1	0	0	1	1.16	1	0	0	0	0	
Ipca Laboratories Ltd.	India	1	1	0	0	0.58	1	0	0	0	0	
Micro Laboratories Ltd.^†^	India	1	0	0	1	0.44	1	0	0	0	0	
											% Substandard:	
Total 12 Manufacturers with WHOPIRs	–	82	39	41	2	0.67	76	6	0	0	7.3%	*P* = 0.0011
Total 146 Manufacturers without WHOPIRs	–	544	123	178	243	1.00	418	82	39	5	23.2%	
Total all 158 Manufacturers of Non-SRA Generics	–	626	162	219	245	0.97	494	88	39	5	21.1%	–

API = active pharmaceutical ingredient; CN price ratio = country-normalized price ratio (see Materials and Methods); DRC = Democratic Republic of the Congo; OOS = out of specification; SRA = stringent regulatory authority; USP = United States Pharmacopeia; WHOPIR = WHO Public Inspection Report. In this study, many medicine samples were collected in health facilities, and it is unknown whether the manufacturers’ storage recommendations were complied with from the time of manufacture until the time of sample collection. Changes in medicine quality may have occurred owing to inappropriate transport and storage conditions, and noncompliance with USP specifications is therefore not necessarily due to substandard manufacturing or packaging. Only one WHO-prequalified medicine, manufactured by Cipla Ltd., India, was found in this study. It was not included in this table, but is in group A of Table 4.

* Significance was calculated for the difference between the proportions of substandard medicines among the 12 manufacturers with WHOPIRs and the 146 manufacturers without WHOPIRs.

^†^ Two further samples from Medopharm Pvt. Ltd. and four further samples from Micro Laboratories Ltd are listed among the falsified medicines in Table 3. These falsifications likely occurred outside the responsibility of these manufacturers.

Notably, the median country-normalized price ratio of the 82 samples from manufacturers with WHOPIRs was 0.67, 31% lower than the median ratio of 0.97 observed for all 626 non-SRA generics (Table 6).

Very few samples produced by any of the manufacturers with WHOPIRs had been collected in our study in Nigeria (Table 6), although nine of these 12 manufacturers were listed with products in Nigeria’s Registered Drug Product Database.^47^ One manufacturer encountered in the present study was among the current producers of WHO-prequalified medicines,^38^ but no WHOPIR was available for it at the WHO websites.^45^^,^^46^ This was Aurobindo Pharma Ltd., India, which was represented only by a single sample (which was not WHO-prequalified itself). That sample showed a moderate deviation in dissolution testing.

### Prices and quality of non-SRA generic medicines from suppliers listed in the MSH International Medical Products Price Guide.

The MSH International Medical Products Price Guide includes a list of 17 suppliers of essential medicines, and it attempted to “ensure that the prices listed in this Guide are from reputable suppliers with established quality assurance processes in place.”^30^^,^^31^ Indeed, in the present study, the 47 medicine samples from suppliers listed in the MSH Guide were found to show a low rate of substandard samples (4.3%), and at the same time, their median price was lower (by 24%) than the median price observed for all non-SRA generics. Most of the 47 samples from suppliers listed in the MSH Guide were found in government and church health facilities in the DRC; none was found in our study in Nigeria.

### Prices and quality of non-SRA generics packaged in blisters or in bulk containers.

In Nigeria, the quality of tablets sold in bulk containers (usually of 1,000 tablets) rather than in blisters was found to be very poor in this study: of 41 samples, four (9.8%) were falsified and 18 (43.9%) were substandard, including six samples with extreme deviations and three further samples with API contents even lower than 50% of the stated amount. At the same time, the price per tablet of the samples sold in bulk containers was only 13.3% cheaper than those sold in blisters. However, the package type had not been recorded in Cameroon and the DRC, and it is yet unclear whether these findings can be generalized.

## DISCUSSION

The present study investigated prices and quality of different medicines in three countries. Without exception, originator brand medicines, which are generally first registered in a country with an SRA, and generic medicines manufactured in countries with SRAs, were found to be of good quality based on the assay and dissolution criteria investigated in the present study. Likewise, Bate et al.^11^^,^^12^ had reported a good quality of originator (innovator) brands collected in LMICs. Also, all SRA generics investigated in this study showed good quality, consistent with the procurement practices of international agencies such as the Global Fund^35^ and the Global Drug Facility,^36^ which consider that medicines with a marketing authorization issued by an SRA can be relied upon and do not need reassessment to be eligible for procurement.^40^

However, the good quality of the originator brands and the SRA generics comes at a price: Within the samples investigated here, the SRA generics were approximately twice as expensive as non-SRA generics, and originator brands were three times as expensive (Figure 4). Similar price differences between originators and non-SRA generics were found in previous studies.^4^^,^^48^ These high prices may be prohibitive for a major part of the population in LMICs, particularly when they do not benefit from any form of health insurance, as well as for governmental and nongovernmental healthcare services in these countries.

In the present study, only one of 711 samples was found to represent a WHO-prequalified medicine. This sample, like the few WHO-prequalified medicines found in an earlier study by our group,^49^ was of good quality, but obviously no general conclusions can be drawn based on these few data. The WHO-prequalified medicines^37^^,^^38^ play a preeminent role in vertical programs addressing, for example, HIV and tuberculosis,^35^^,^^36^ but less so in organizations working in primary healthcare and hospitals.^50^ An increased market share of WHO-prequalified medicines in LMICs would be desirable but would require an expansion of the scope of the WHO prequalification program as well as a commitment of purchasers to prioritize WHO-prequalified medicines.

As shown in Figure 4, generic medicines manufactured in countries without SRAs were clearly more affordable than originator brands or SRA generics. However, of these non-SRA generics, 21.1% were found to be substandard in assay, dissolution, or both. Notably, no relevant differences were seen in this study between branded and unbranded non-SRA generics regarding price or quality. This is in striking contrast to the situation in India and Indonesia, where local manufacturers frequently market the same pharmaceutical product in two different versions, a branded and an unbranded one.^18^^,^^19^ Advertising efforts by the manufacturer are focused on the branded version, which is sold at a much higher price than the unbranded one. For the non-SRA generics, such a practice was not observed in the three African countries investigated in the present study. However, among the 45 generics from countries with an SRA, the median country-normalized price ratio of the branded ones (*n =* 21) was 2.3 times higher than that of the unbranded ones (*n =* 24). All 45 samples complied with pharmacopeial specifications. Kaplan and Wirtz^42^ had named the investigation of the prices of branded versus unbranded generics in LMICs as a research priority, and the present study may help to fill this research gap.

Of all non-SRA generic samples investigated in this study, 51.5% had been manufactured in India and 22.2% in China. In terms of different brands/products, 48.2% of non-SRA generic products had been manufactured in India and 22.1% in China. This confirms the well-known strong position of these two countries on the African pharmaceutical market. In African countries, both politicians and members of the general public frequently express the expectation that a substitution of medicine imports from India and China by locally produced medicines would improve medicine quality and reduce medicine prices.^51^^–^^53^ The data shown in Figure 6 suggest that this may not automatically be the case: Within the samples investigated in this study, medicines produced in African countries showed, overall, neither better quality nor lower prices than those imported from India and China. Additional capacity building will be required to further develop local manufacturing practices in African countries. To improve economies of scale, access of the individual African manufacturers to the entire African market is likely to be important. Unfortunately, the present study indicated only a low extent of intra-African cross-border pharmaceutical trading. This fact may in part result from deliberate import prohibitions^54^^,^^55^ and may be a topic to be addressed by the forthcoming African Medicines Agency.^56^

Among the 711 samples investigated in this study, 626 were non-SRA generics. These were from 158 different manufacturers in 16 different countries and included 132 (21.1%) substandard samples. The key challenge for quality assurance in local medicine procurement is how to identify manufacturers and suppliers that provide good-quality medicines at an affordable cost. It might be speculated that medicines offered for cheap prices would be of lower quality or, conversely, that medicines of better quality would necessarily be expensive. However, as shown in Figure 7, in Supplemental Figure 1, and in Supplemental Table 5, within the non-SRA generics investigated in this study there was no positive correlation between medicine price and medicine quality. Cheaper non-SRA generic medicines did not show lower quality than more expensive ones; therefore, higher medicine prices are apparently not explained by higher investment in quality assurance.

The two studies by Bate et al. reported that substandard medicines were, on average, 13.6–18.7%^11^ or 10%^12^ cheaper than good-quality medicines. However, originator brands manufactured in countries with an SRA had been included in this comparison; therefore, the results of Bate et al. are not in conflict with those of the present study.

Of all 626 non-SRA generics, 21.1% were substandard. A significantly lower percentage of substandard medicines (7.3%; Table 6) was found for the combined samples from the 12 manufacturers for which Public Inspection Reports (WHOPIRs) had been published as part of the WHO Medicine Prequalification Program.^45^^,^^46^ The WHO prequalification is granted to a specific product by a specific manufacturer; therefore, publication of the WHOPIR is not an official endorsement of that manufacturer by the WHO, and some of the archived WHOPIRs^46^ may be outdated. Nevertheless, the results of the present study show that products coming from manufacturers with WHOPIRs are likely to include fewer substandard samples than those of other manufacturers. At the same time, the median price of medicines from the manufacturers with WHOPIRs was even lower (by 33%) than that of the other non-SRA generics (Table 6). These findings indicate that adequate quality assurance does not necessarily imply an increase in medicine prices.^57^^,^^58^ Supplemental Table 6 lists the 98 manufacturers currently with WHOPIRs. Of these, 91 are located in non-SRA countries. However, currently this list contains only four manufacturers from sub-Saharan Africa, and an increase in this number would be desirable.

Further possibilities to try to identify manufacturers and suppliers with adequate quality systems may certainly exist (e.g., through the QUAMED database of audited manufacturers and distributors, accessible for a fee)^59^ and through individual supplier qualification, according to the WHO model quality assurance system for procurement agencies.^60^ Local stakeholders who can and want to procure internationally may also consult the European Commission DG ECHO Register of Humanitarian Procurement Centers^61^ (which also includes three suppliers located in sub-Saharan Africa).

It is noteworthy that, without exception, all medicine samples representing originator brands or SRA generics, as well as nearly all medicines from manufacturers with WHOPIRs, were found to comply with USP specifications for both assay and dissolution. In Cameroon and the DRC, most of them had been collected in retail drug outlets or health facilities in different regions or provinces, including rural areas far from urban centers. According to the WHO guidelines for stability testing of pharmaceuticals,^62^ these two countries belong to the climatic zones IVb (hot and very humid) and IVa (hot and humid), respectively. It appears likely that many of these samples were exposed to inadequate transport and storage conditions prior to their collection, but nevertheless they retained their good quality. On the other hand, in Nigeria medicines were collected on the wholesale level, often directly from the manufacturers; nevertheless, they showed a high percentage of substandard samples. This suggests that, at least for the medicines investigated in the present study, medicine quality was primarily determined by the quality of manufacturing and the quality of the packaging, whereas transport and storage conditions have been of only secondary importance. Conclusive evidence from future studies investigating both the quality of medicines from different sources and the transport and storage conditions of these samples would be desirable.

Among the 711 medicine samples investigated in this study, 14 (2.0%) were identified as falsified medicines (Table 3). Originator brands, SRA generics, and non-SRA generics were all affected by falsification. Of 72 originator brands and SRA generics, two were falsified (see Table 3), representing a 2.8% risk to the consumer to purchase a falsified originator or SRA product. Obviously, very different actions are required to curb the proliferation of falsified medicines (arising from fraudulent intent), compared with substandard medicines (arising mainly from nonintentional shortcomings in their manufacturing and packaging). The price range of the falsified medicines was similar to that of the non-SRA generics (Figure 4), consistent with the results of Bate et al.^11^^,^^12^

### Limitations of this study.

Although this study is, to the best of our knowledge, the most comprehensive investigation of the relationship of medicine prices and quality in LMICs published so far, it has clear limitations that may be appropriately addressed in future research. This study was conducted in only three countries of sub-Saharan Africa, and the results cannot be generalized to other countries or continents. Chemical investigation of medicine quality was restricted to assay and dissolution testing and did not include testing for related substances or further quality criteria. The study included only a limited number of originator brands and SRA generics; therefore, the ratio of the prices of these categories of medicines compared with that of non-SRA generics may result differently if more or other originator brands and SRA generics are investigated. Generic medicines stated to be produced in countries with an SRA were considered SRA generics in this study, without confirming whether indeed a marketing authorization had been issued by the respective SRA for that medicine. Although this study included medicine samples collected both on the wholesale and retail levels (or in health facilities), the levels investigated were not identical in the three countries. Multi-country studies investigating prices and quality of the same types of medicines in the same types and levels of collection sites would be desirable, and funding for such (expensive) studies should be made available. The most recent MSH reference prices used in this study were from 2015.^30^^,^^31^ In this study, the medicine samples from Cameroon and the DRC (*n =* 459) were purchased in 2017/2018 and the included medicine samples from Nigeria (*n =* 252) in 2021/2022. This is 2–3 years, or 6–7 years, after publication of the most recent MSH reference guide. Supplemental Table 7 shows the MSH reference prices for the medicines investigated in this study for the years 2010–2015. The price differences between different APIs (or API combinations) remain reasonably stable over the years, suggesting that a use of newer reference prices (if available) would not have significantly altered the price ratios to the MSH reference prices calculated in this study. Nevertheless, the unavailability of more recent reference prices is a serious limitation for medicine price investigations employing the WHO/HAI methodology,^32^ and an update of the MSH International Medical Products Price Guide or the creation of a similar reference is an urgent priority for global health research.

## Supplemental Materials

10.4269/ajtmh.24-0309 Supplemental Materials

## Data Availability

A replication dataset for this paper (including the underlying data and the analysis code in SPSS format) is available in the pharmRχiv repository: https://doi.org/10.57747/pharmrxiv-2024071830145-000.
