# Supplementary material for: Relationship between Prices and Quality of Essential Medicines from Different Manufacturers Collected in Cameroon, the Democratic Republic of the Congo, and Nigeria
Source: Am J Trop Med Hyg. 2024 Oct 8;111(6):1378–95. doi: 10.4269/ajtmh.24-0309 (PMC11619510; doi:10.4269/ajtmh.24-0309)
Supplement: Supplemental Materials [file tpmd240309.SD1.pdf]

Supplementary Material for

Relationship between prices and quality of essential medicines from different manufacturers collected in Cameroon, the Democratic Republic of Congo, and Nigeria

Julia Gabel,[1] Difäm-EPN Minilab Network,[2] Peter Martus,[3] and Lutz Heide[1*]

[1] Pharmaceutical Institute, Eberhard Karls University Tuebingen, Tuebingen, Germany.
[2] The individuals from this group are listed in the Acknowledgments.
[3] Institute for Medical Biometrics and Clinical Epidemiology, University Hospital Tübingen, Tübingen, Germany.

Content:

Supplementary Figure S1. Comparison of prices of in-specification and substandard samples of non-SRA generic medicines. 2

Supplementary Table S1. Numbers of samples in different medicine categories found in different types of collection sites. 3

Supplementary Table S2: Numbers of medicine samples in different quality categories 4

Supplementary Table S3: Comparison of prices between medicine categories by one-factorial ANOVA with pairwise comparisons using Tukey's (B) test. 6

Supplementary Table S4: Prices and quality of generic medicines manufactured in different non-SRA countries. 7

Supplementary Table S5: Logistic regression analysis of the association between medicine prices and medicine quality. 8

Supplementary Table S6: Manufacturers of finished pharmaceutical products for whom a WHO Public Inspection Report is available on the respective WHO websites. 9

Supplementary Table S7: MSH reference prices for the medicines investigated in this study for the years 2010-2015 12

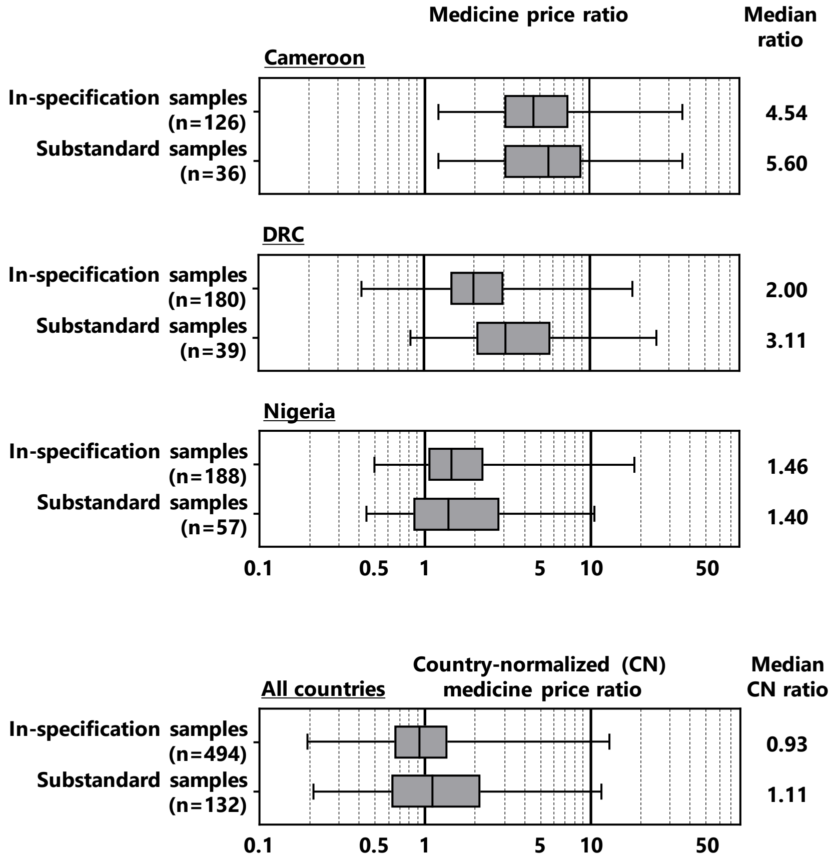

Supplementary Figure S1. Comparison of prices of in-specification and substandard samples of non-SRA generic medicines. Scatterplots of the quantitative results of each assay and dissolution analysis against the price of each medicine sample are shown in Figure 7 in the main manuscript.

Supplementary Table S1. Numbers of samples in different medicine categories found in different types of collection sites.

	Originator	SRA generic	WHO-prequalified product	Branded non-SRA generic	Unbranded non-SRA generic	Falsified medicine	Total
**Cameroon**							
Government health facilities	0	0	0	6	29	0	**35**
Church health facilities	0	3	0	15	40	4	**62**
Private pharmacies	14	28	1	5	12	0	**60**
Informal vendors	0	5	0	18	37	4	**64**
**Total Cameroon**	**14**	**36**	**1**	**44**	**118**	**8**	**221**
**DRC**							
Government health facilities	0	0	0	4	24	1	**29**
Church health facilities	1	2	0	22	40	0	**65**
Private pharmacies	6	5	0	46	31	0	**88**
Informal vendors	2	1	0	29	23	1	**56**
**Total DRC**	**9**	**8**	**0**	**101**	**118**	**2**	**238**
**Nigeria**							
Licensed vendors	1	0	0	76	30	0	**107**
Markets	1	1	0	79	60	4	**145**
**Total Nigeria**	**2**	**1**	**0**	**155**	**90**	**4**	**252**
**Total three countries**	**25**	**45**	**1**	**300**	**326**	**14**	**711**

Supplementary Table S2: Numbers of medicine samples in different quality categories

	In speci-fication	Moderate deviation	Extreme deviation	<50% of declared API amount	falsified
**Medicine quality in different types of collection sites (Main manuscript, Figure 3)**					
**Cameroon:**					
Government health facilities (n=35)	27	5	3	0	0
Church health facilities (n=62)	48	7	3	0	4
Private pharmacies (n=60)	58	1	1	0	0
Informal vendors (n=64)	44	6	10	0	4
**DRC:**					
Government health facilities (n=29)	25	3	0	0	1
Church health facilities (n=65)	61	4	0	0	0
Private pharmacies (n=88)	71	14	3	0	0
Informal vendors (n=56)	40	14	1	0	1
**Nigeria**					
Licensed vendors (n=107)	82	16	7	2	0
Markets (n=145)	109	18	11	3	4
**Medicine quality in different categories of medicines (Main manuscript, Figure 4)**					
**Cameroon:**					
Originator (n=14)	14	0	0	0	n.a.
SRA generic (n=36)	36	0	0	0	n.a.
WHO-prequalified product (n=1)	1	0	0	0	n.a.
Branded non-SRA generic (n=44)	38	6	0	0	n.a.
Unbranded non-SRA generic (n=118)	88	13	17	0	n.a.
Falsified medicine (n=8)	n.a.	n.a.	n.a.	n.a.	8
**DRC:**					
Originator (n=9)	9	0	0	0	n.a.
SRA generic (n=8)	8	0	0	0	n.a.
WHO-prequalified product (n=0)	no WHO prequalified products sampled				
Branded non-SRA generic (n=101)	81	18	2	0	n.a.
Unbranded non-SRA generic (n=118)	99	17	2	0	n.a.
Falsified medicine (n=2)	n.a.	n.a.	n.a.	n.a.	2
**Nigeria**					
Originator (n=2)	2	0	0	0	n.a.
SRA generic (n=1)	1	0	0	0	n.a.
WHO-prequalified product (n=0)	no WHO prequalified products sampled				
Branded non-SRA generic (n=155)	116	23	11	5	n.a.
Unbranded non-SRA generic (n=90)	72	11	7	0	n.a.
Falsified medicine (n=4)	n.a.	n.a.	n.a.	n.a.	4
**All countries:**					
Originator (n=25)	25	0	0	0	n.a.
SRA generic (n=45)	45	0	0	0	n.a.
WHO-prequalified product (n=1)	1	0	0	0	n.a.
Branded non-SRA generic (n=300)	235	47	13	5	n.a.
Unbranded non-SRA generic (n=326)	259	41	26	0	n.a.
Falsified medicine (n=14)	n.a.	n.a.	n.a.	n.a.	14

See next page for legend.

Supplementary Table S2 (continued): Numbers of medicine samples in different quality categories

	In speci-fication	Moderate deviation	Extreme deviation	<50% of declared API amount	falsified
**Quality of generic medicines manufactured in different non-SRA countries (Main manuscript, Figure 6)**					
**Collected in Cameroon:**					
Nigeria (n=1)	1	0	0	0	n.a.
Kenya (n=0)	no samples found				
Other African countries (n=18)	12	4	2	0	n.a.
India (n=90)	73	10	7	0	n.a.
China (n=45)	32	5	8	0	n.a.
Other Asian countries (n=4)	4	0	0	0	n.a.
**Collected in DRC:**					
Nigeria (n=0)	no samples found				
Kenya (n=20)	17	3	0	0	n.a.
Other African countries (n=19)	15	4	0	0	n.a.
India (n=110)	84	23	3	0	n.a.
China (n=69)	63	5	1	0	n.a.
Other Asian countries (n=1)	1	0	0	0	n.a.
**Collected in Nigeria**					
Nigeria (n=96)	68	18	6	4	n.a.
Kenya (n=0)	no samples found				
Other African countries (n=0)	no samples found				
India (n=117)	90	15	12	0	n.a.
China (n=23)	21	1	0	1	n.a.
Other Asian countries (n=3)	3	0	0	0	n.a.
**All countries:**					
Nigeria (n=97)	69	18	6	4	n.a.
Kenya (n=20)	17	3	0	0	n.a.
Other African countries (n=37)	27	8	2	0	n.a.
India (n=317)	247	48	22	0	n.a.
China (n=137)	116	11	9	1	n.a.
Other Asian countries (n=8)	8	0	0	0	n.a.

See methods for definition of quality categories. For "falsified medicines", no reliable information is available about the name and location of their manufacturer, therefore they were placed into a separate category, and not assigned to a country of manufacturing. Further explanations are given in the legends of Figures 3, 4 and 6 in the main manuscript.

n.a., not applicable.

Supplementary Table S3: Comparison of prices between medicine categories by one-factorial ANOVA with pairwise comparisons using Tukey's (B) test.

ANOVA

Log10 of country-normalized medicine price ratio

	Sum of Squares	df	Mean Square	F	Sig.
Between Groups	14,483	3	4,828	46,658	<,001
Within Groups	71,603	692	,103		
Total	86,086	695			

Post Hoc Tests

Log10 of country-normalized medicine price ratio

Tukey B[a,b]

Four medicine categories for data analysis	N	Subset for alpha = 0.05		
		1	2	3
unbranded non-SRA generic	326	-,0089		
branded non-SRA generic	300	,0563		
SRA generic	45		,2546	
Originator	25			,7302

Means for groups in homogeneous subsets are displayed.

a. Uses Harmonic Mean Sample Size = 58,289.

b. The group sizes are unequal. The harmonic mean of the group sizes is used. Type I error levels are not guaranteed.

For this analysis, the prices were logarithmically transformed (basis 10).

Supplementary Table S4: Prices and quality of generic medicines manufactured in different non-SRA countries.

	**Total**	Median country-normalized price ratio	in specification	moderate deviation	extreme deviation	<50% of the declared API amount
Benin	**5**	1.40	3	0	2	0
Burundi	**1**	1.40	1	0	0	0
Cameroon	**5**	0.80	3	2	0	0
Ghana	**5**	0.67	3	2	0	0
Kenya	**20**	1.10	17	3	0	0
DRC	**9**	0.94	5	4	0	0
Nigeria	**97**	1.03	69	18	6	4
Togo	**3**	0.80	3	0	0	0
Uganda	**9**	1.09	9	0	0	0
**Total Africa**	**154**	**1.05**	**113**	**29**	**8**	**4**
China	**137**	0.93	116	11	9	1
Hongkong	**3**	3.26	3	0	0	0
India	**317**	0.88	247	48	22	0
Malaysia	**2**	5.66	2	0	0	0
Sultanate of Oman	**1**	1.17	1	0	0	0
Thailand	**1**	1.29	1	0	0	0
Turkey	**1**	1.01	1	0	0	0
**Total Asia**	**462**	**0.93**	**371**	**59**	**31**	**1**
Country not stated	**10**	1.29	10	0	0	0
**Grand Total**	**626**	**0.97**	**494**	**88**	**39**	**5**

Falsified medicines were not included into this table since no reliable information is available on their country of manufacturing. Also, the single WHO-prequalified medicine found in this study (manufactured in India) was not included. For some countries of manufacturing, price and quality information is based only on a single sample.

Supplementary Table S5: Logistic regression analysis of the association between medicine prices and medicine quality.

**Medicine categories**	n	Regression coefficient B	Standard error	Wald test	df	**Sig. (*p* value)**	Exp(B)[1]	95% C.I. for Exp(B)		Odds[1] ratio	95% C.I.for odds ratio	
								Lower	Upper		Lower	Upper
**All samples**	711	0.098	0.262	0.139	1	**0.710**	1.103	0.659	1.844	1.02	0.92	1.13
**All non-falsified samples**	697	0.200	0.270	0.551	1	**0.458**	1.222	0.720	2.074	1.04	0.94	1.16
**All non-SRA generics**	626	0.750	0.301	6.189	1	**0.013**	2.116	1.172	3.820	1.16	1.03	1.31
**Branded non-SRA generics**	300	0.480	0.411	1.363	1	**0.243**	1.617	0.722	3.621	1.10	0.94	1.29
**Unbranded non-SRA generics**	326	1.073	0.453	5.611	1	**0.018**	2.923	1.203	7.101	1.24	1.04	1.48

Binary logistic regression was applied to investigate the association between the logarithmically transformed (basis 10) country-normalized price ratio and the quality of medicines (dichotomized; in-specification = 0, substandard =1).

[1] "Exp(B)" is the odds ratio for a ten-fold increase of the country-normalized price ratio. The "Odds ratio" shown on the right-hand side is the odds ratio for a two-fold increase of the country-normalized price ratio. (Example: for unbranded non-SRA generics, doubling of the country-normalized price ratio increases the risk of the medicine sample being substandard by the factor of 1.24.) This odds ratio is calculated as the fifth root of Exp(B).

A logistic regression model including medicine categories as predictor and quality as outcome did not converge, due to the observation of "null cells" for originator medicines and SRA generics.

A replication dataset including the underlying data and the analysis code in SPSS format is available in the pharmRχiv repository, https://pharmrxiv.de/receive/pharmrxiv_mods_00020723, and allows to access further outputs of this logistic regression analysis.

Supplementary Table S6: Manufacturers of finished pharmaceutical products for whom a WHO Public Inspection Report is available on the respective WHO websites.

WHO Public Inspection Reports are available at the WHO websites https://extranet.who.int/prequal/inspection-services/who-public-inspection-reports-whopirs-medicines and https://extranet.who.int/prequal/inspection-services/prequalification-reports/whopirs-archive); the information at these websites was accessed on May 7, 2024.

	Manufacturer	Country
1	Acme Formulation Pvt Ltd	India
2	Agila Specialties Private Limited	India
3	Aizant Drug Research Solutions Pvt Ltd	India
4	Ajanta Pharma Limited	India
5	Alkem Laboratories Ltd	India
6	Anhui Biochem Bio-Pharmaceutical Co Ltd	China
7	Aspen Port Elizabeth (Pty) Ltd	South Africa
8	AXIS Clinicals	United States of America
9	Bayer de México S.A. de C.V.	Mexico
10	Beijing Novartis Pharma Co Ltd	China
11	Beximco Pharmaceutical Ltd	Bangladesh
12	Bio Pharma Services	Canada
13	Cadila Pharmaceuticals Ltd	India
14	China Resources Zizhu Pharmaceutical Co Ltd	China
15	Cipla Limited	India
16	Dean Superior Textile Co Ltd	China
17	Dong-A ST Co Ltd	Republic of Korea
18	Dr. Reddy's Laboratories Ltd	India
19	Egyptian International Pharmaceutical Industries Company (EIPICO)	Egypt
20	Eisai Pharmatechnology and Manufacturing Pvt Ltd	India
21	Emcure Pharmaceuticals Ltd	India
22	European Egyptian Pharmaceutical Industries (EEPI)	Egypt
23	Famy Care Ltd	India
24	Farmak JSC	Ukraine
25	Getz Pharma Pvt Ltd	Pakistan
26	Grindeks JSC	Latvia
27	Guilin Pharmaceutical Co Ltd	China
28	Hainan Poly Pharma Co Ltd	China
29	HBM Pharma s.r.o.	Slovakia
30	Hetero Labs Limited (Unit-V)	India
31	Hisun Pharmaceuticals (Hangzhou) Co Ltd	China
32	HLL Lifecare Ltd	India
33	Immacule Lifesciences Pvt Ltd	India
34	Indoco Remedies Limited	India
35	Instituto de Tecnologia em Fármacos (Farmanguinhos)	Brazil
36	Ipca Laboratories Ltd	India
37	Jai Pharma Ltd	India
38	Joint Stock Company JSC "Biocom"	Russian Federation
39	Joint-Stock Company "Halychpharm"	Ukraine
40	KBN-Zhejiang Pharmaceutical Co	China
41	Laboratorios Leon Farma	Spain

42	Laurus Labs Limited	India
43	Livzon (Group) Pharmaceutical Factory	China
44	Lupin Ltd	India
45	Macleods Pharmaceuticals Ltd	India
46	Maphar Laboratories	Morocco
47	Meditab Specialities Pvt Ltd	India
48	Medopharm Private Limited	India
49	Mepro Pharmaceuticals Pvt Ltd	India
50	Merck S. A. de C.V. (Naucalpan de Juárez)	Mexico
51	Micro Labs Ltd	India
52	Milan Laboratories (India) Pvt Ltd	India
53	Mission Vivacare Limited	India
54	MSN Laboratories Pvt Ltd	India
55	Mylan Laboratories Ltd	India
56	North China Pharmaceutical Co Ltd	China
57	Novartis Pharmaceuticals Corporation	United States of America
58	Oxalis Labs	India
59	PharmEvo (Pvt) Ltd	Pakistan
60	PT Caprifarmindo Laboratories	Indonesia
61	PT Kalbe Farma Tbk	Indonesia
62	PT SANBE FARMA Sterile Preparations Plant	Indonesia
63	PT Tunggal Idaman Abdi	Indonesia
64	Qilu Pharmaceutical Co Ltd	China
65	Quality Chemical Industries Ltd (QCIL)	Uganda
66	Questa Care Ltd	Kenya
67	Ranbaxy Laboratories Limited	India
68	Remington Pharmaceutical Industries (Pvt) Ltd	Pakistan
69	Renata Limited	Bangladesh
70	S Kant Healthcare Limited	India
71	Sandoz Private Limited	India
72	Sandoz SA (Pty) Ltd	South Africa
73	Shanghai Dahua Pharmaceutical Co	China
74	Shanghai Desano Bio-Pharmaceutical Co Ltd	China
75	Shanghai Harvest Pharmaceutical Co Ltd	China
76	Shasun Pharmaceuticals Limited	India
77	Sinopharm Zhijun (Shenzhen) Pharmaceutical Co Ltd	China
78	Square Pharmaceuticals Ltd (SPL)	Bangladesh
79	Stada Vietnam Joint Venture Co Ltd	Vietnam
80	Steril-Gene Life Sciences Pvt Ltd	India
81	Strides Arcolab Ltd	India
82	Strides Pharma Science Limited	India
83	Strides Shasun Ltd	India
84	Suheung Co Ltd	Republic of Korea
85	Sun Pharmaceutical Industries Ltd	India
86	Svizera Labs Pvt Ltd	India
87	Technolog Private Joint Stock Company (Group of pharmaceutical companies Lekhim)	Ukraine
88	The ACME Laboratories Ltd	Bangladesh
89	The Government Pharmaceutical Organization (GPO)	Thailand
90	The Jordanian Pharmaceuticals Manufacturing Company	Jordan

91	UAB Santonika	Lithuania
92	Universal Corporation Ltd	Kenya
93	Zhejiang Apeloa Kangyu Pharmaceutical Co Ltd	China
94	Zhejiang Hisun Pharmaceutical Co Ltd	China
95	Zhejiang Holley Nanhu Pharmaceutical Co Ltd	China
96	Zhejiang Huahai Pharmaceutical Co Ltd	China
97	Zhejiang Jiangbei Pharmaceutical Co Ltd	China
98	Zydus Lifesciences Limited	India

Supplementary Table S7: MSH reference prices for the medicines investigated in this study for the years 2010-2015

Active pharmaceutical ingredient (API)	Dosage form	Strength [mg]	MSH reference price (US Cent/unit)					
			2010	2011	2012	2013	2014	2015
Amoxicillin	tablet/ capsule	500	2.90	3.19	3.13	3.13	3.11	3.00
Amoxicillin/ clavulanic acid	tablet	500/125	18.69	22.44	19.61	20.14	13.50	16.41
Atenolol	tablet	50	0.95	1.02	1.06	1.18	1.03	1.07
Ceftriaxone	powder for inject.	1000	69.00	65.86	70.41	58.87	74.70	39.80
Cefuroxime axetil	tablet	500	39.58	38.71	37.83	36.37	55.03	39.11
Chloroquine	tablet	250	0.95	1.10	1.07	1.26	1.40	1.37
Ciprofloxacin	tablet	500	3.06	3.28	3.78	4.18	4.30	3.73
Co-trimoxazole	tablet	400/80	1.06	1.11	1.13	1.19	1.24	1.20
Dexamethasone	tablet	0.5	0.42	0.49	0.52	0.64	0.80	0.70
Doxycycline	tablet/ capsule	100	1.17	1.23	1.44	1.44	1.41	1.33
Fluconazole	tablet/ capsule	150	6.79	6.13	6.75	4.78	7.92	6.92
Furosemide	tablet	40	0.43	0.48	0.61	0.58	0.67	0.61
Glibenclamide	tablet	5	0.34	0.42	0.42	0.67	0.68	0.57
Hydrochloro-thiazide	tablet	50	0.45	0.53	0.50	0.52	0.52	0.49
Metformin	tablet	500	1.05	1.79	1.68	1.78	1.69	1.50
Metronidazole	tablet	250	0.52	0.59	0.61	0.62	0.64	0.61
Penicillin V	tablet	250	1.63	1.01	1.58	1.64	1.72	1.76
Salbutamol	tablet	4	0.29	0.30	0.32	0.40	0.34	0.32

The most recent MSH reference prices, used in this study, are from 2015 (Main Manuscript, ref. 30). In this study, the medicine samples from Cameroon and the DRC (n=459) were purchased in 2017/18, and the included medicine samples from Nigeria (n=252) in 2021/22. This table shows that the price differences between different APIs (or API combinations) remain reasonably stable over the years, suggesting that a use of newer reference prices (if available) would not have significantly altered the price ratios to the MSH reference prices calculated in this study.
